# Selectively Fluorinated PAMAM–Arginine Conjugates as Gene Delivery Vectors

**DOI:** 10.1021/acs.bioconjchem.3c00139

**Published:** 2023-05-23

**Authors:** Carola Romani, Paola Gagni, Mattia Sponchioni, Alessandro Volonterio

**Affiliations:** † Department of Chemistry, Materials and Chemical Engineering “Giulio Natta”, Politecnico di Milano, Via Mancinelli 7, 20131 Milano, Italy; ‡ Istituto di Scienze e Tecnologie Chimiche “Giulio Natta”-SCITEC, CNR, Via Mario Bianco 9, Milan 20131, Italy

## Abstract

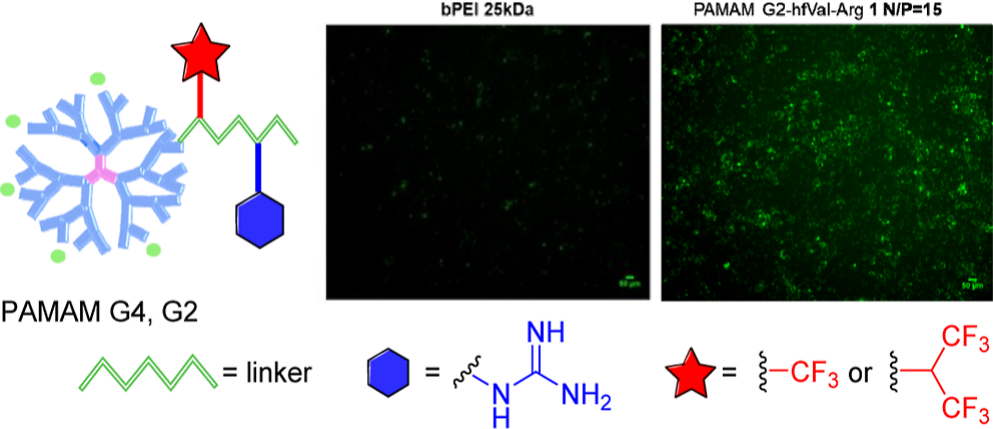

Polyamidoamine (PAMAM) dendrimers are among the most studied cationic polymers as non-viral gene delivery vectors. However, an “ideal” PAMAM-based gene delivery vector is still missing due to the high manufacturing costs and non-negligible cytotoxicity associated with the use of high-generation dendrimers, whereas low-generation dendrimers are far from displaying efficient gene transfection. In order to cover this gap in the literature, in this study, we propose the functionalization of the outer primary amines of PAMAM G2 and PAMAM G4 with building blocks bearing fluorinated moieties along with a guanidino functional group. We have designed and synthetized two fluorinated arginine (Arg)-based Michael acceptors which were straightforwardly “clicked” to PAMAM dendrimers without the need for coupling reagents and/or catalysts. The obtained conjugates, in particular, derivative **1** formed starting from the low-cost PAMAM G2 and a building block bearing two trifluoromethyl groups, were able to efficiently complex plasmid DNA, had negligible cytotoxicity, and showed improved gene transfection efficiency as compared to undecorated PAMAM dendrimers and a corresponding unfluorinated PAMAM–Arg derivative, with derivative **1** being two orders of magnitude more efficient than the gold standard branched polyethylenimine, bPEI, 25 kDa. These results highlight the importance of the presence of trifluoromethyl moieties for both gene transfection and a possible future application in ^19^F magnetic resonance imaging.

## Introduction

The discovery that the main role of DNA is the long-term storage of genetic information has driven the great scientific achievements of the twentieth century.^1^ In fact, DNA and RNA are gaining increasing attention as possible therapeutic agents, due to their potentiality for the treatment, at the genetic level, of diseases that nowadays are still untreatable, such as cancer, monogenic diseases, muscular dystrophy, and neurodegenerative and cardiovascular diseases.^1−6^

To obtain the biological effects, the therapeutic sequence of nucleic acids must be transported to the target site (cells or tissues), overcoming different intracellular and extracellular barriers, such as the inability to cross the cell membrane due to the presence of negatively charged phosphate groups and their hydrophilicity and the susceptibility to endogenous nuclease degradation.^2,7^ For this reason, to safely and efficiently deliver the therapeutic genes to the target cells, along with the use of modified viruses, which is still nowadays the most efficient strategy with its pros and cons,^8^ in the last few decades, a variety of innovative, chemically based vectors, such as non-viral cationic lipids and polymeric vectors, have been proposed in the literature. Concerning cationic polymers, branched polyethylenimine (bPEI) 25 kDa and high-generation polyamidoamine dendrimers (PAMAM) are considered the gold standard of polycationic transfecting agents because of their high transfection efficiency, mainly related to the high amine density.^3,7−10^ However, the high charge density is also the main responsibility of the non-negligible cytotoxicity of these compounds.^1,7^

In particular, PAMAMs were mostly investigated for their great potential in biomedical applications, as they are tunable in size, chemical structure, in terminal group functionalization.^11^ An ethylenediamine core and terminal primary amines characterize the dendritic structure of the polymer. Successive branching called generations (Gs) increase the size, doubling the positively charged primary amines on the surface, resulting not only in higher transfection efficiencies (TEs) but also in more severe cytotoxicity. This cytotoxicity/TE tradeoff makes the research of the right balance the most crucial challenge to be overcome.^12^ In this scenario, two main strategies have been explored. The first one relies on shielding terminal primary amines and increasing the cellular uptake through chemical functionalization of high-generation dendrimers with amino acids, including arginine (Arg),^13−15^ alkyl chains,^16^ peptides (such as RGD and SRL),^17,18^ PEGylated chains,^19,20^ and aminoglycosides,^21−23^ among others. However, the high manufacturing cost, the expensive reagents required for high-generation PAMAM syntheses, the overcompression of genetic materials observed at a high generation that could affect TE,^24^ and cytotoxicity issues have driven to seek a different approach. Indeed, the functionalization of low-generation PAMAM, such as PAMAM-G2, to mimic the higher generations in terms of TEs, maintaining their intrinsic low cytotoxicity, is taking hold. Cross-linking or functionalization strategies could be exploited to increase the cationic surface and obtain a more stable and flexible polymeric structure.^25−29^ Among the different functionalizations, the addition of biocompatible moieties, such as amino acids like Arg, characterized by having a guanidinium group,^30^ and/or the introduction of fluorinated moieties have gained interest as strategies to decrease the vector cytotoxicity and to enhance TE, avoiding gene cargo compression.^31,32^

Specifically, fluorination seems to enhance some important properties of non-viral vectors, such as DNA condensation at a lower polymer amine/DNA phosphate group (N/P) ratio and serum resistance due to the non-reactivity of fluorinated moieties.^31,33^ Furthermore, it has been shown that fluorinated moieties tethered to polymeric structures improve cellular uptake and avoid opsonization issues, due to the hydrophobic and bio-inert behavior of fluorinated added components.^34,35^

Moreover, the introduction of equivalent fluorine atoms (isotope ^19^F) could also be considered a novel strategy to enhance quantitative magnetic resonance imaging (MRI).^36,37^ Indeed, MRI has become the principal noninvasive diagnostic investigation tool for various clinical problems, acquiring information about functions inside the living organism, in both health and disease conditions. The use of fluorine in MRI overcomes the drawbacks associated with the use of ^1^H-MRI, such as the background signal derived from tissues and water and the long acquisition times and the dosage and toxicity issues associated with the use of contrast agents. For this reason, commercially available fluorinated molecules have been used as the ^19^F probe, such as hexafluorobenzene (HFB), perfluorononane (PFN), and perfluorocarbon (PFC).^36−38^

However, some difficulties in gene cargo release due to excessive amount of fluorine atoms and the problems encountered when trying to develop fluorinated compounds characterized by favorable chemical and biological properties, like low cytotoxicity, high biocompatibility, long shelf life, simple manufacturing process, and suitable and measurable functionalization degree, still remain significant obstacles to overcome.

**Chart 1:**
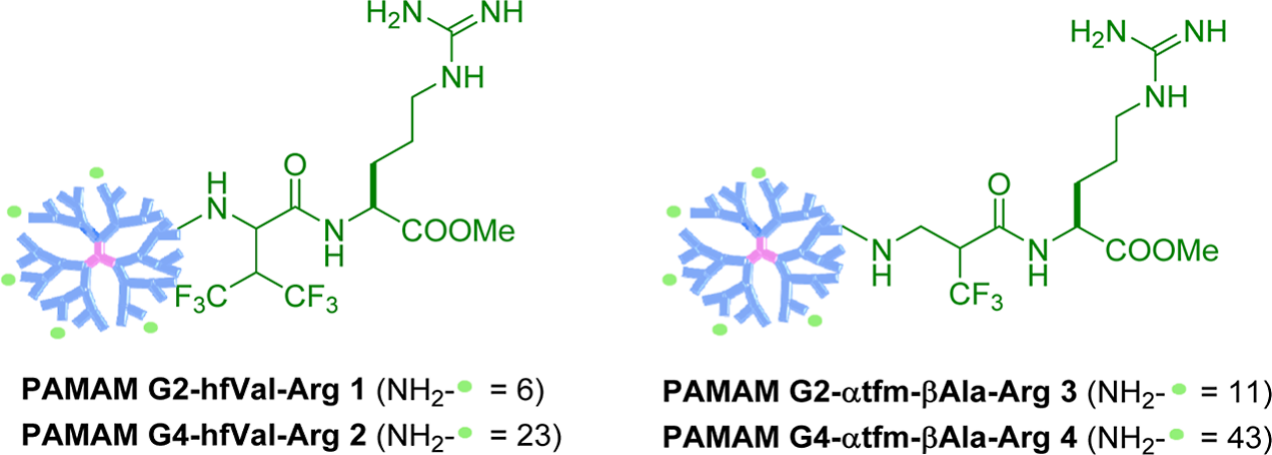
Structures of Selectively Fluorinated PAMAM–Arg Conjugates **1–4**

In this work, we explore for the first time new combined synthetic strategies to obtain four novel, selectively fluorinated PAMAM–Arg conjugates, namely, PAMAM G2 and G4 tethered to hexafluorovaline-Arg dipeptide (hfVal-Arg) **1** and **2**, respectively, and PAMAM G2 and G4 tethered to α-trifluoromethyl-β-alanine-Arg dipeptide (α-tfm-β-Ala-Arg) **3** and **4**, respectively, as safe and efficient vectors for gene delivery (Chart 1). Fluorinated multifunctional building blocks derived from Arg have been designed, synthetized, and efficiently used in PAMAM functionalization. All the obtained building blocks are characterized by specific and proper functional groups, in particular, (i) the guanidinium group, crucial for cellular membrane interactions and the consequent internalization of the polymer/nucleic acids complexes, (ii) a highly electrophilic carbon–carbon double bond, suitable for the straightforward functionalization of the PAMAM dendrimers through Michael addition or anti-Michael addition, and (iii) a fluorinated moiety bearing a single or two different trifluoromethyl (tfm) groups that are becoming increasingly attractive for novel fluorinated building block synthesis as biologically active compounds.^39,40^

The in vitro cytotoxicity and TE of these novel non-viral vectors have been evaluated, showing that these conjugates are more efficient than the materials recognized as gold standards, specifically undecorated PAMAM G4 and PEI 25 kDa, with the PAMAM G2–hfVal-Arg **1** derivative being the most performing conjugate. These results pave the way for the use of these conjugates, in particular, PAMAM G2 derivatives, associated with lower manufacturing costs, as multifunctional drug/gene delivery systems with the possible implementation in ^19^F MRI applications.

## Results and Discussion

### Synthesis of Fluorinated PAMAM–Arg Conjugates

**Scheme 1:**
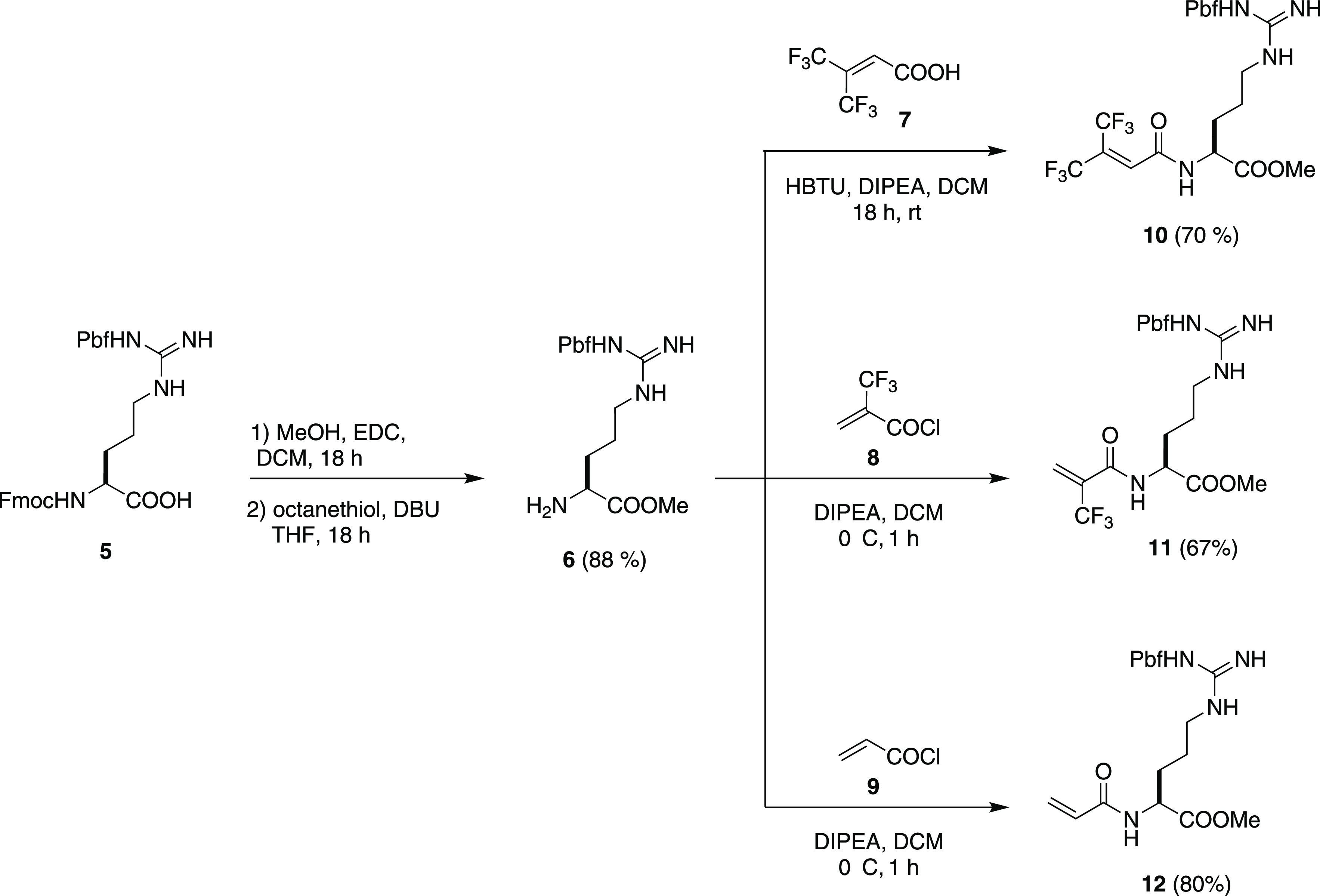
Synthesis of Fluorinated and Unfluorinated Arg-Michael Acceptors **10–12** (EDC = Ethyl-Diisopropylcarbodiimide; DBU = 1,5-diazabiciclo(5.4.0)undec-7-ene; HBTU = (2-(1*H*-benzotriazol-1-yl)-1,1,3,3-tetramethyluronium Hexafluorophosphate; DIPEA = *N*,*N*-Diisopropylethylamine; and DCM = Dichloromethane)

In the past years, we have developed a straightforward strategy for the synthesis of peptidomimetics incorporating a fluorinated moiety relying on the efficient Michael addition of α-aminoester to α,β-unsaturated carbon–carbon double bonds bearing a highly electronegative tfm group which increases the reactivity of the electrophilic alkene.^41,42^ Since the reaction is fast, clean, and does not require coupling reagents, we thought to exploit it in the “click” functionalization of the terminal primary amino groups of PAMAM dendrimers with ad hoc synthetized fluorinated Michael acceptors. Accordingly, we designed two different fluorinated Arg-derived building blocks having specific functional groups for absolving different purposes: (i) an electrophilic carbon–carbon double bond suitable for the “click” functionalization through Michael addition (or anti-Michael addition) of PAMAM dendrimers, (ii) a fluorinated moiety bearing a single or two different trifluoromethyl groups, which will increase the reactivity of the intermediates and could be exploited for molecular fluorine tracking and imaging purposes through ^19^F-MRI,^36,37^ and (iii) the guanidino functional group belonging to arginine that would increase the transfection efficiency of the final PAMAM vectors. Fluorinated Arg-Michael acceptors **10** and **11** were prepared in high yields starting from Arg(Pbf)-OMe **6**, obtained by esterification and Fmoc-deprotection of commercially available Fmoc-Arg(Pbf)-OH **5**, by coupling with commercially available 4,4,4-trifluoro-3-(trifluoromethyl) crotonic acid **7** or acylation with α-(trifluoromethyl)acryloyl chloride **8**,^42^ respectively (Scheme 1).

Analogously, we decided to synthetize the corresponding unfluorinated Arg-Michael acceptor **12** to explore the influence of the trifluoromethyl groups on the “click” functionalization of the PAMAM dendrimers and on the cytotoxicity/TE of the final PAMAM conjugates.

**Scheme 2:**
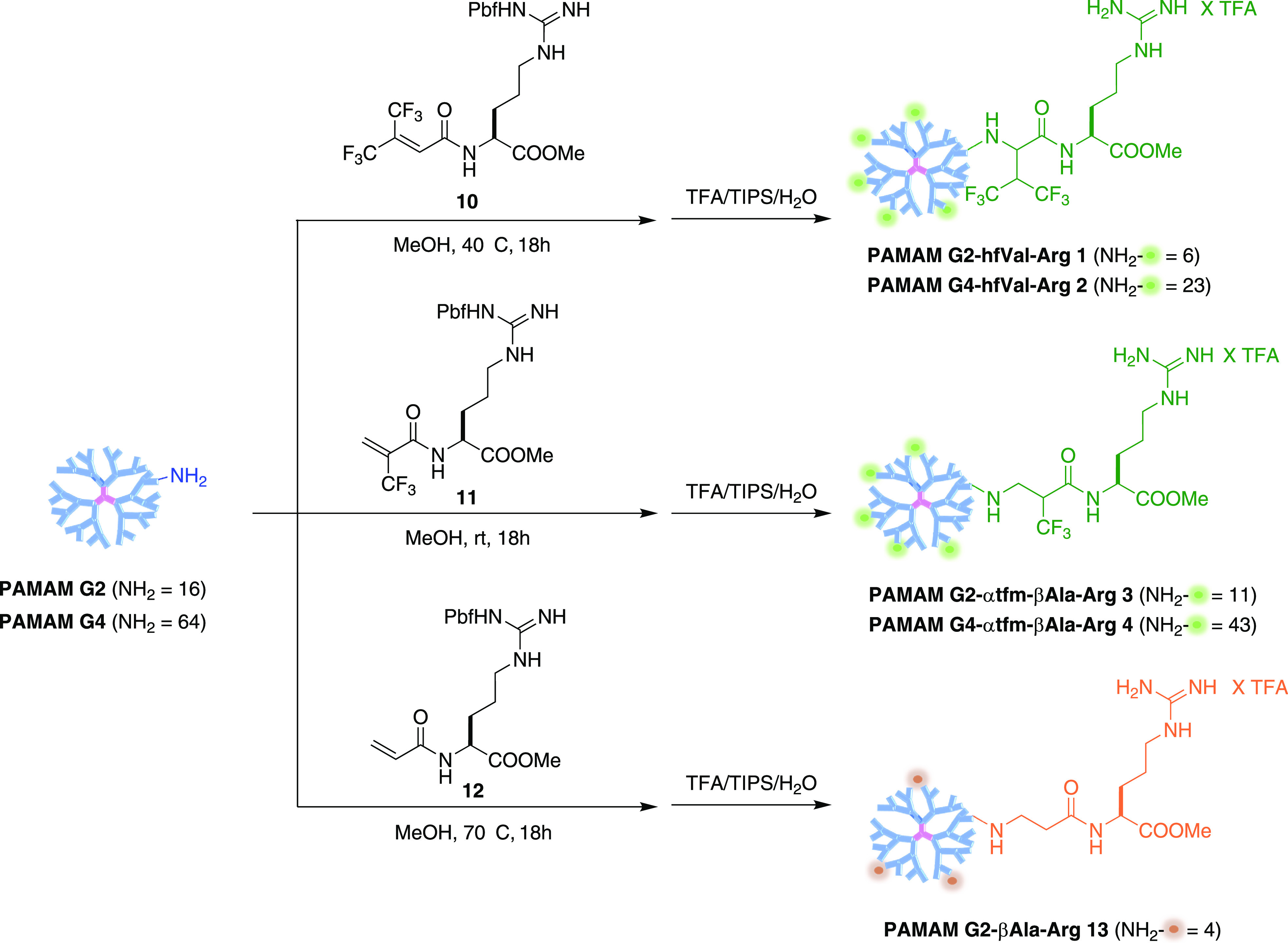
Synthesis of Fluorinated PAMAM-Arg Derivatives **1–4** and Unfluorinated PAMAM G2-Arg Derivative **13** (TFA = Trifluoroacetic Acid and TIPS = Triisopropyl Silane)

Click functionalization of PAMAM G2 and PAMAM G4 was performed smoothly in MeOH as the solvent at different temperatures depending on the reactivity of the Michael acceptors (Scheme 2). Indeed, derivative **10** having two trifluoromethyl groups in β-position reacted at 40 °C through an anti-Michael mechanism, giving rise to the formation of PAMAM G2–hfVal-Arg **1** and PAMAM G4–hfVal-Arg **2** with 37.5 and 36.0% functionalization degrees (FDs), respectively. Derivative **11**, being much more reactive due to a single trifluoromethyl group in α-position, reacted well at room temperature, providing PAMAM G2-αtfm-βAla-Arg **3** and PAMAM G4-αtfm-βAla-Arg **4** with 65.6 and 66.5% FDs, respectively. The lower FDs obtained with *bis*-trifluoromethyl Michael acceptor **10** are probably due to both its lower reactivity and the higher sterical hindrance of the conjugate obtained. As expected, to have a comparable FD, with unfluorinated derivative **12**, we had to run the reaction at 70 °C. Nonetheless, we could isolate PAMAM G2-βAla-Arg **3** with only 4 primary amines functionalized out of the 16 (25.0% FD), confirming that the presence of the fluorinated moieties in Michael acceptors **10–11** makes such compounds much more reactive.

**Figure 1:**
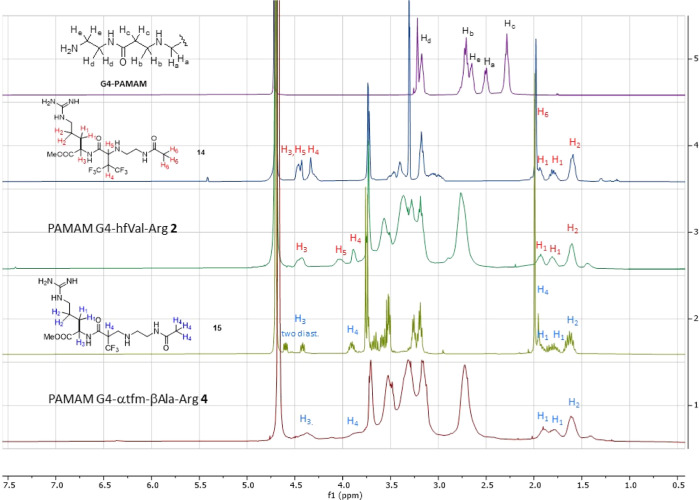
^1^H NMR spectra recorded in D_2_O of PAMAM G4 (violet spectrum), model compound **14** (light blue spectrum), PAMAM G4–hfVal-Arg **2** (olive spectrum), model compound **15** (green spectrum), and PAMAM G4−αtfm-βAla-Arg **4** (red spectrum).

According to our previous studies,^21−23^ the FDs were established by ^1^H NMR, integrating signals belonging to the PAMAM dendrimer *versus* signals belonging to the Arg-containing arm. In order to select the suitable signals, we synthetized model compounds **14–16** by reacting *N*-(2-aminoethyl)acetamide with the Michael acceptors **10–12** (Scheme S1, Supporting Information). In Figure 1, the ^1^H NMR spectra of PAMAM G4 (violet spectrum), model compound **14** prepared by the addition of *N*-(2-aminoethyl)acetamide to **10** (light blue spectrum), PAMAM G4–hfVal-Arg **2** (olive spectrum), model compound **15** prepared by the addition of *N*-(2-aminoethyl)acetamide to **11** (green spectrum), and PAMAM G4-αtfm-βAla-Arg **4** (red spectrum), all recorded in D_2_O, are represented as an example.

As highlighted in the spectra of PAMAM G4–Arg conjugates **2** and **4**, there are two sets of protons that can be used to quantify the Arg-arms that are tethered to the PAMAM dendrimers, namely, the protons belonging to the two methylene groups of the Arg side chain (protons H_1_ and H_2_) that are the only protons resonating in the region between 1.5 and 2.0 ppm and the proton H_3_ tethered to the α-carbon of Arg amino acid, which resonates at the lowest field (around 4.5 ppm). These protons can be integrated *versus* the protons resonating between 2.6 and 2.8 ppm belonging to the methylene group in α position to the carbonyl groups in PAMAM dendrimers (protons Hc that are 56 for PAMAM G2 and 248 for PAMAM G4). Indeed, as highlighted in the spectrum of the model compounds **14** and **15**, there are no signals belonging to the arm in this range of chemical shift. The chemical characterization comprising the chemical shift of the ^19^F NMR signals and the calculated molecular weights (MWs) of all the dendrimers synthesized are summarized in Table 1.

**Table 1:**
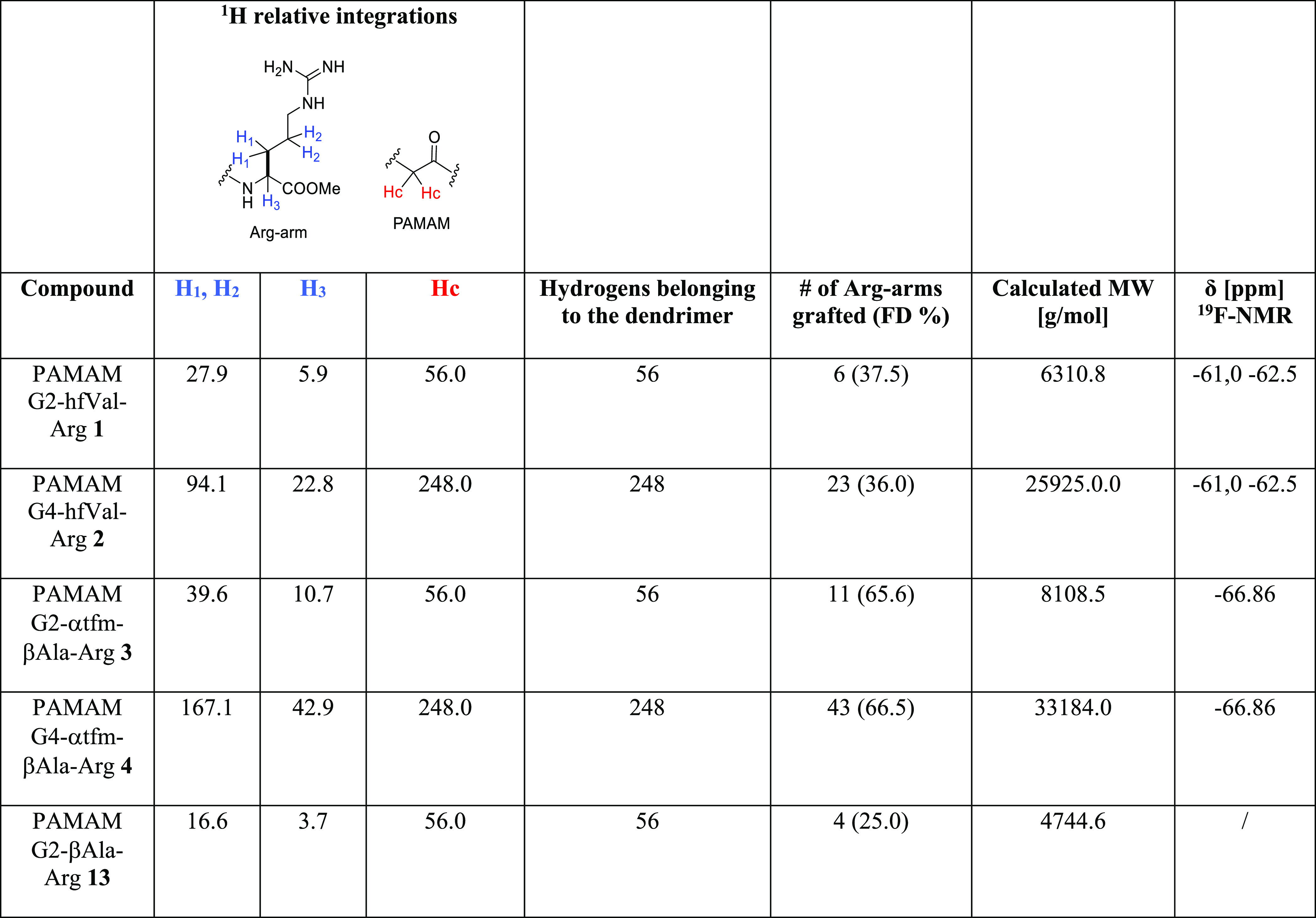
Chemical Characterization of PAMAM–Arg Conjugates **1–4** and **13**

### Biophysical Characterization of Fluorinated PAMAM-Based Dendriplexes

**Figure 2:**
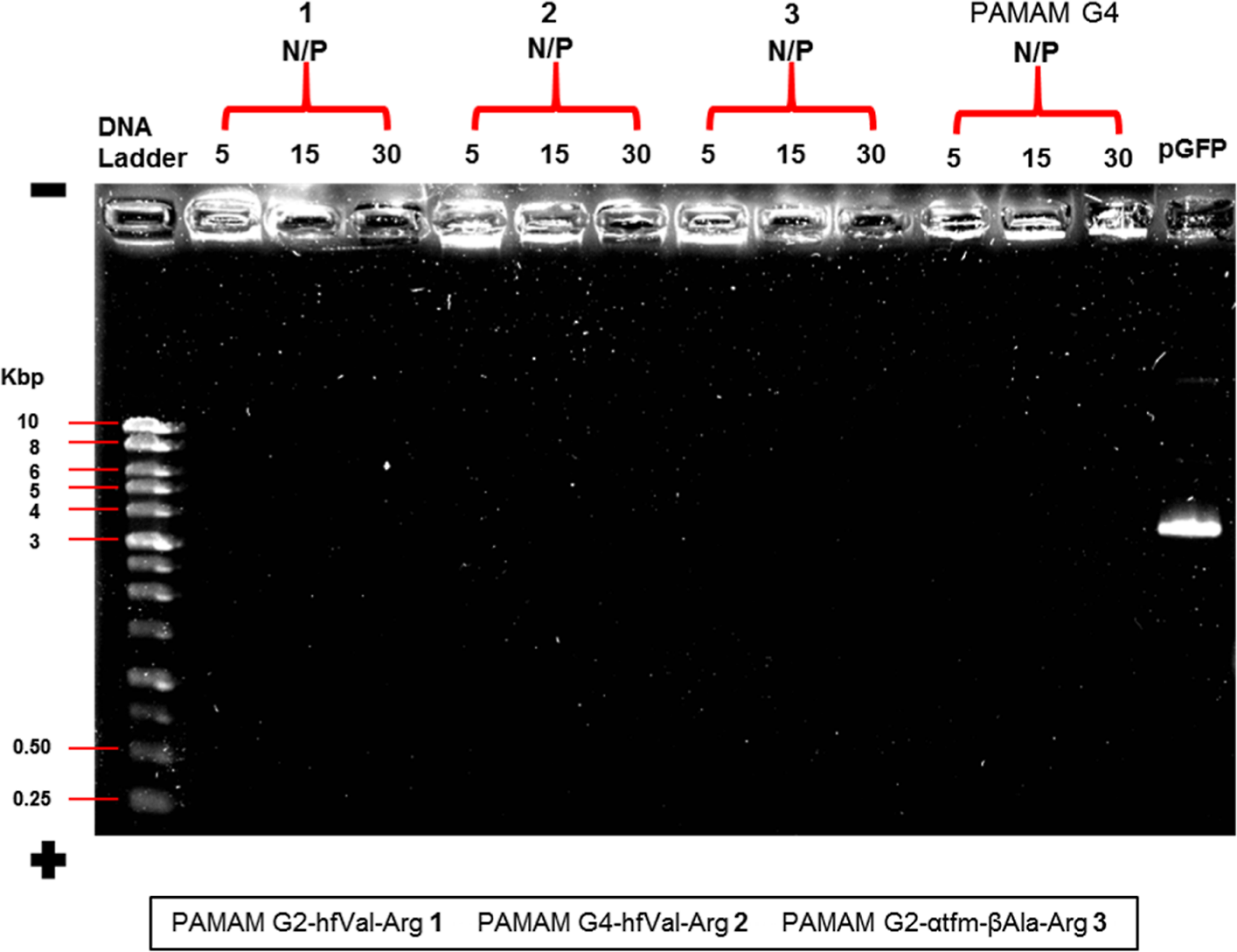
Agarose gel analysis of the plasmid complexed by cationic fluorinated dendrimers at different N/P ratios is reported: twelve complexes derived from four dendrimers (PAMAM G2–hfVal-Arg **1**, PAMAM G4–hfVal-Arg 2, PAMAM G2−αtfm-βAla-Arg 3, and undecorated PAMAM G4) at different N/P ratios were loaded. In the first and in the fourteenth well, DNA ladder and free DNA were loaded, respectively. The stain of DNA with Diamond dye indicates the good complexation of the synthesized system, whose complexed DNA, showed as bright fluorescent blot, remains in the loaded wells, whereas free DNA migration along the gel is observed.

Complexation studies and size analysis were performed *via* agarose gel retardation and DLS, respectively. Agarose gel retardation assay was used to evaluate the complexation ability of fluorinated PAMAM–Arg conjugates **1–4** and **13**. The proof-of-concept was previously demonstrated by using the herring sperm, as a model non-therapeutic DNA, complexed with a proper solution of the dendrimers in deionized water, at N/P ratios equal to 5, 15, and 30. Once defined the optimal experimental conditions and stated that the assayed dendrimers were able to complex DNA as compared to free nucleic acid (Figure S1, Supporting Information), the target plasmid of green fluorescent protein (pGFP) was tested with the most performing PAMAM G2–hfVal-Arg **1**, PAMAM G4–hfVal-Arg 2, and PAMAM G2−αtfm-βAla-Arg 3 dendrimers, at N/P ratios equal to 5, 15, and 30 each (Figure 2) and compared to undecorated PAMAM G4, used as the positive control. In all cases, we detected the formation of complexes big enough to retard DNA migration as compared to free DNA (last lane). Moreover, fluorinated dendrimers **1–3** exhibit plasmid complexation ability also at a low N/P ratio as compared to the positive control. The highly intense signals detectable in wells demonstrate that complexed DNA remains stuck in the gel wells during migration, testifying the achievement of a good dendritic complexation.

The ability to form complexes and their stability were also confirmed by analysis of the hydrodynamic diameter (*D*_H_), polydispersity index (PDI) by DLS, and ξ-potential with and without gene cargo (Table 2).

**Table 2:** Intensity-Weighted Mean Hydrodynamic Size (*D*_H_), PDI, and ξ-potential of the Fluorinated Dendrimers Obtained without (w/o) and with (w/) the Genetic Cargo at N/P 15

sample	*D*_H_ w/o [nm]	PDI w/o [-]	ξ-potential w/o [mV]	*D*_H_ w/[nm]	PDI **w/[-]**	ξ-potential w/[mV]
PAMAM G2–hfVal-Arg **1**	398 ± 4	0.41 ± 0.12	30 ± 0.75	188 ± 6	0.19 ± 0.01	25 ± 0.56
PAMAM G4–hfVal-Arg **2**	300 ± 4	0.41 ± 0.01	31 ± 1.51	200 ± 6	0.19 ± 0.01	25 ± 1.53
PAMAM G2−αtfm-βAla-Arg **3**	350 ± 2	0.18 ± 0.06	29 ± 2.42	250 ± 15	0.20 ± 0.03	24 ± 0.58
PAMAM G4−αtfm-βAla-Arg **4**	450 ± 3	0.61 ± 0.12	31 ± 3.05	350 ± 10	0.35 ± 0.02	29 ± 2.52

As an indication of the formation of DNA/PAMAM complexes, Table 2 highlights the reduction in the size of the dendriplexes compared to their non-complexated counterparts. In fact, when a nucleic acid is added, the positive charges of functionalized dendrimers interact with the negative ones present in the nucleic acid, enforcing the complexation favored by the presence of fluorine atoms, compacting the core, and reducing the size of the dendriplexes. Accordingly, we measured a reduction on the ξ-potential of the dendrimers before and after complexation with the plasmid. Taking together the biophysical analysis results, the balanced amounts of fluorine atoms present inside PAMAM G2–hfVal-Arg **1** and PAMAM G4–hfVal-Arg **2** structures permit to avoid the issues due to the “fluorous effect”,^43,44^ overcoming the strongest fluorine–fluorine interaction, which could lead to hydrophobic aggregate formation, blocking gene cargo entrance and consequent electrostatic interactions with the positive charges of the dendritic structure. On the other hand, an excess of fluorine interactions and possible steric hindrance due to the higher presence of functionalizing building blocks on the surface of PAMAM G4-αtfm-βAla-Arg **4** could prevent an effective reduction of the size, as a result of only partial complexation of the genetic cargo at a low N/P ratio. For higher N/P ratios, PAMAM G4-αtfm-βAla-Arg **4** seems to be able to complex the genetic material, but some problem in gene cargo release is observed during transfection studies *in vitro* (see below).

### *In Vitro* Cytotoxicity and Transfection Efficiency of pDNA/Fluorinated PAMAM-Arg Complexes

PAMAM dendrimers are one of the most studied cationic polymers in the field of gene delivery. However, they suffer from the so-called TE/cytotoxicity paradox which limits their application. Indeed, the TE and cytotoxicity of PAMAMs are strictly correlated to their generation: the greater the generation, the higher the TE but, at the same time, the higher the cytotoxicity.

**Figure 3:**
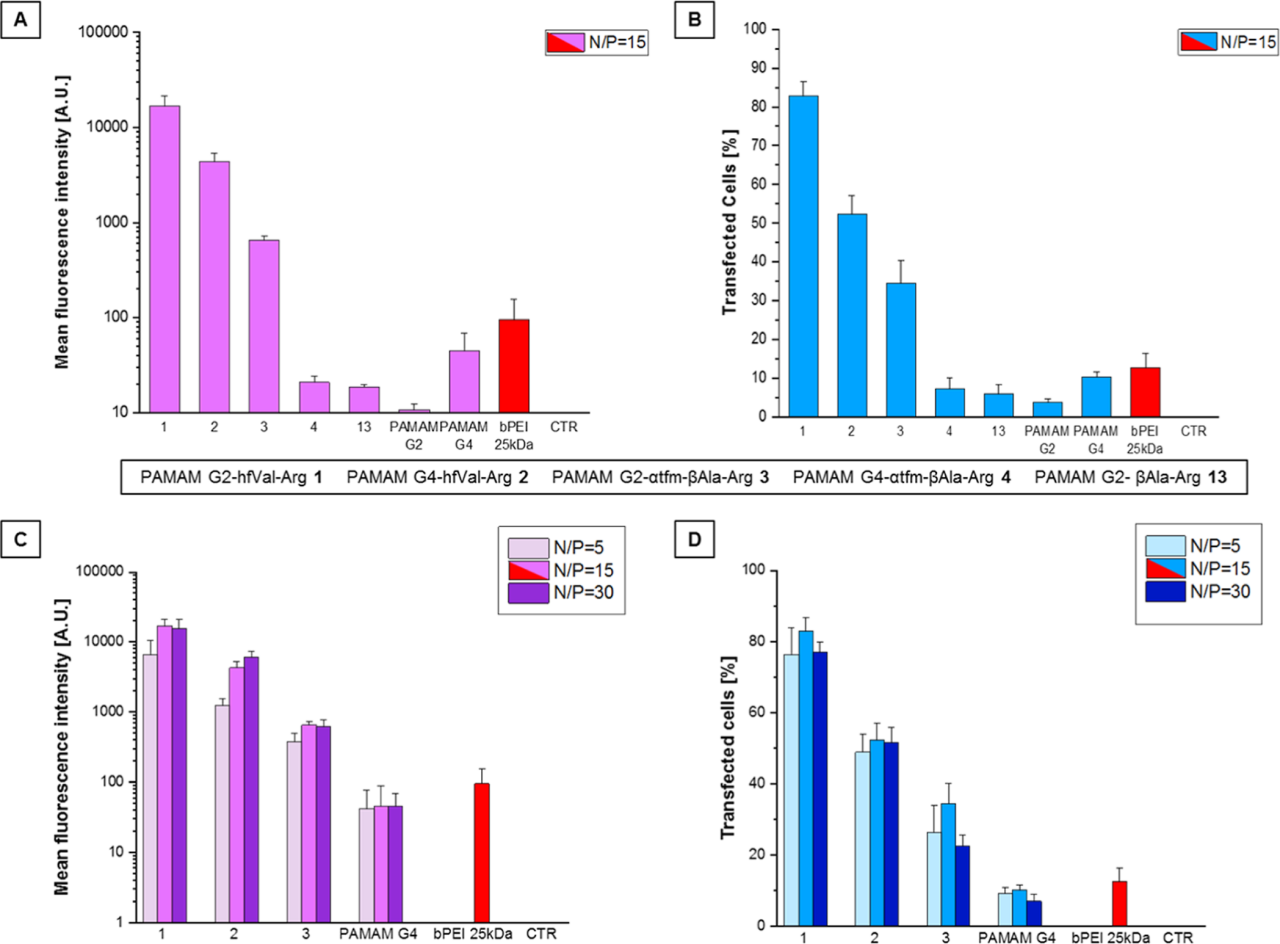
TEs of fluorinated PAMAM–Arg conjugates **1–4** compared to unfluorinated PAMAM G2-βAla-Arg **13** and to positive controls PAMAM G4 and bPEI 25 kDa (N/P fixed to 15). TE tests at an N/P ratio of 15 as a function of mean fluorescence intensity (A) and percentage of transfected cells (B) and TEs of the most performing PAMAM–Arg conjugates at different N/P ratios as a function of mean fluorescence intensity (C) and percentage of transfected cells (D) measured *via* ImageJ software after 48 h. For negative control (CTR), HUVECs without the transfecting agent were considered. Error bars are standard deviation, and statistical significance is *p* < 0.001 for all dendrimers compared to positive control bPEI 25 kDa.

The TEs and cytotoxicity of the four fluorinated PAMAM synthesized **1–4** and the unfluorinated PAMAM G2-βAla-Arg **13** were investigated and compared to undecorated PAMAM G2 and PAMAM G4 and to bPEI 25 kDa as the positive control. pGFP and human umbilical vein endothelial cells (HUVECs) as a model primary cell system were used for transfection efficiency and cytotoxicity evaluation of the dendrimers. HUVECs are endothelial cells that play an important role in cellular targeting for cardiovascular disease, control of vascular function, and angiogenesis. This cellular line is so strictly linked to the potential use of gene therapy to treat cardiovascular disease, angiogenesis, and spark cellular proliferation inhibition against vasculo-angiogenesis in tumor growth.^5,45^ However, the transfection of endothelial cells is still difficult to achieve, due to the resistance of endothelial cells and the extreme cytotoxic effect of the current non-viral vectors on this cellular line.^46,47^ First, we determined the most efficient conjugates by performing transfection experiments at N/P = 15 (Figure 3A,B). Interestingly, we observed a remarkably increased TE of conjugates PAMAM G2–hfVal-Arg **1**, PAMAM G4–hfVal-Arg **2**, and PAMAM G2−αtfm-βAla-Arg **3** compared to undecorated PAMAM G4 and even to the golden standard bPEI 25 kDa. It is worth noting that all the fluorinated derivatives but PAMAM G4−αtfm-βAla-Arg **4** are also much more efficient than unfluorinated PAMAM G2−βAla-Arg **13**, underlying the importance of the trifluoromethyl groups together with the guanidino moieties.

For the best performing fluorinated dendrimers, TEs at N/P equal to 5 and 30 were also investigated, by evaluating the number of transfected cells and GFP fluorescence intensity following the same procedure used for N/P equal to 15 (Figure 3C,D). As expected, the extent of transfection for each fluorinated PAMAM-Arg derivative was affected by the N/P ratio. PAMAM G2–hfVal-Arg **1** and PAMAM G4–hfVal-Arg **2** showed a higher number of transfected cells (>75 and >50%, respectively) with respect to the bPEI 25 kDa (<20%) at different N/P ratios, suggesting a stronger cellular membrane interaction, due to the guanidinium groups, and their consequent internalization reinforced by fluorine atoms present along the dendritic structure. In fact, the hydrophobic behavior of trifluoromethyl groups favor the phospholipid bilayer crossing, without disrupting the cellular membrane. Furthermore, the higher gene cargo release ability of these conjugates deduced from the obtained results could be considered a proof of no “fluorous effect”, due to a dispersive distribution of trifluoromethyl groups along the surface, without minimizing fluorine atom amount and avoiding fluorine–fluorine interactions. At lower N/P ratios, the presence of bulky, hydrophobic hexafluoroisopropyl groups on the surface of low-generation PAMAM G2–hfVal-Arg **1** could facilitate the gene release to a more extent than in the high-generation PAMAM G4 derivative. Nevertheless, at a higher N/P ratio (N/P = 30), the dendriplexes featured a slight decrease in the transfection efficiency, probably due to a stronger electrostatic interaction so that cells are less able to disassemble them intracellularly. A good transfection ability is also observed for PAMAM G2−αtfm-βAla-Arg **3**, with respect to bPEI 25 kDa. The lower TE observed for PAMAM G4−αtfm-βAla-Arg **4** could be probably ascribed to the larger size of the dendriplexes and to the low complexation ability of the system observed during the biophysical characterization studies, which hinder the internalization of the complexes as well as the amount of pDNA delivered. This in turn may be related to the shape of the building block used for the functionalization and to its steric hindrance between the building block chains along the dendritic surface, limiting plasmid electrostatic interactions, and/or to the increment of a homogenic density of fluorine atoms along the dendritic surface, resulting in a possible “fluorous effect”.^43,44^

**Figure 4:**
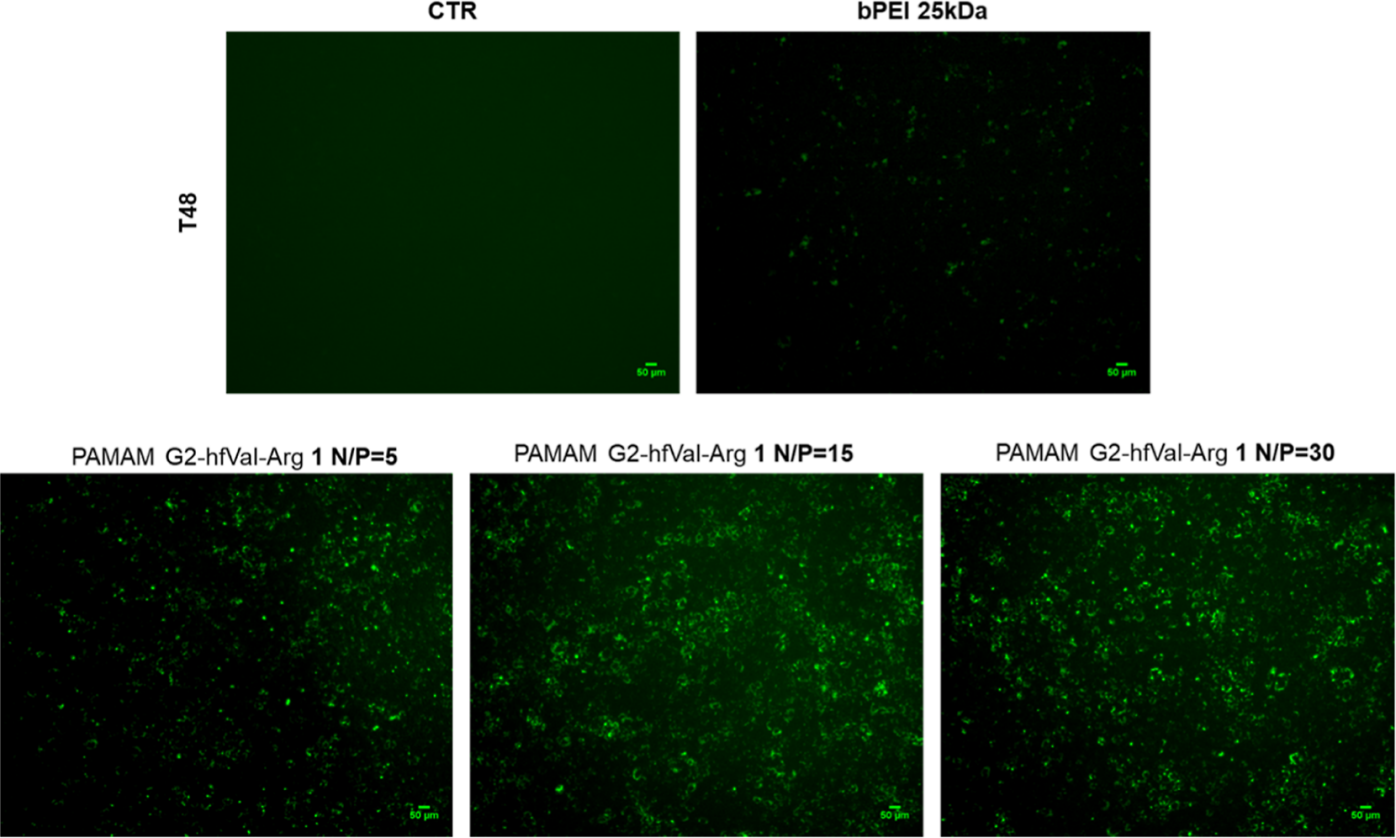
Transfected cell micrographs acquired with Celena S microscopy (filter cube GFP) after 48 h (T48) from dendriplexes added to HUVEC cultures. The images depict cell cultures treated with PAMAM G2–hfVal-Arg **1** at different N/P ratios compared to bPEI 25 kDa; the emitted green fluorescence is related to the GFP expressed by the transfected cells. For the negative control (CTR), HUVECs without the transfecting agent were considered.

For the most performing PAMAM G2–hfVal-Arg **1**, Figure 4 shows the fluorescence micrographs of the cells transfected at N/P 5, 15, and 30 compared to the cells treated with bPEI 25 kDa at N/P = 15 or to the control. The density and intensity of fluorescent cells are clearly higher for **1**/pDNA than bPEI/pDNA regardless the N/P, with N/P = 15 being the best conditions, confirming that PAMAM G2–hfVal-Arg **1** is a valuable gene delivery vector despite the low generation of the PAMAM precursor.

**Figure 5:**
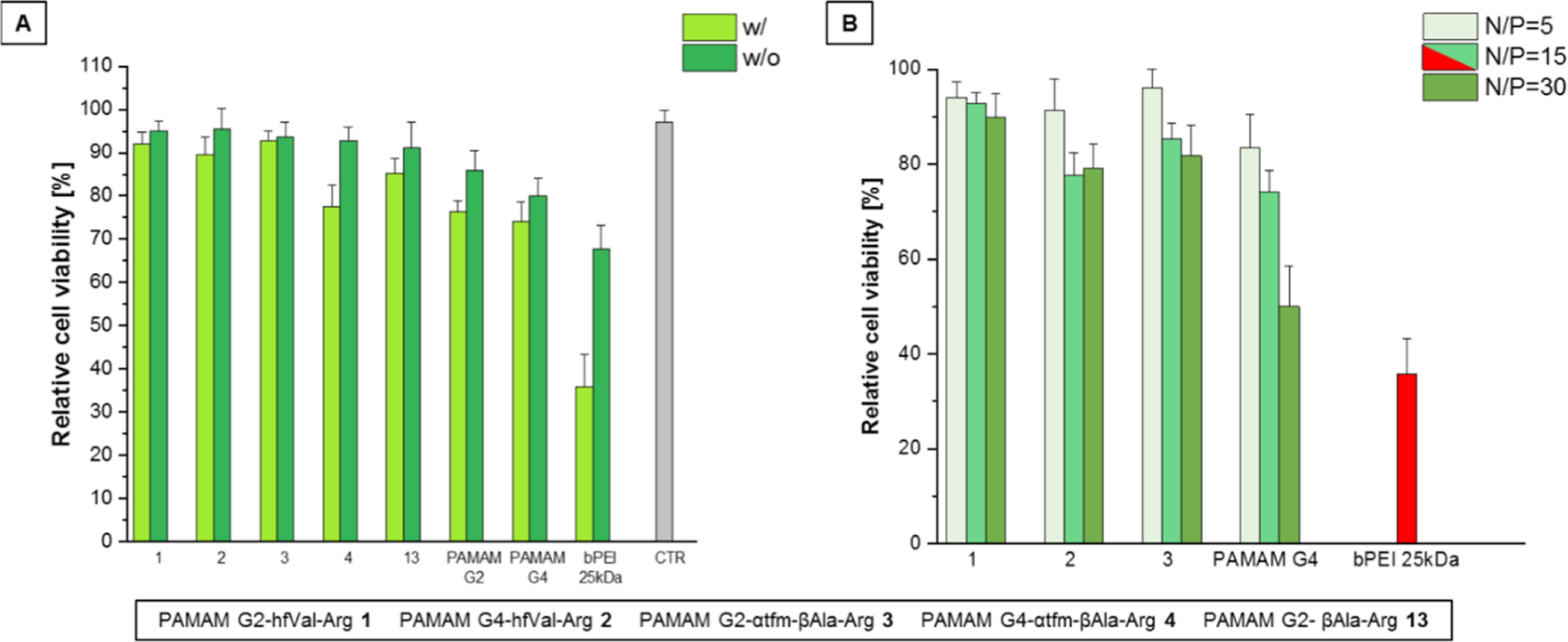
Cytotoxicity w/o and w/ gene cargo expressed as cell viability relative to the control for all synthesized PAMAMs (Figure A) at N/P fixed to 15; cytotoxicity at different N/P ratios for the best performing fluorinated PAMAM–Arg conjugates **1–3** (Figure B).

The second critical attribute for judging on the relevance of a gene delivery system is cytotoxicity. This was studied for PAMAM-Arg conjugates **1–4** and **13** with and without the genetic cargo and compared to the cytotoxicity of undecorated PAMAM G2 and G4 dendrimers and bPEI 25 kDa in the same condition (Figure 5A). The results show that all the PAMAM–Arg conjugates have lower cytotoxicity than bPEI 25 kDa, with **1–3** showing higher cell viability even with respect to their undecorated counterparts. Moreover, the cytotoxicity of the polyplexes is similar to those of the free polymers, indicating a not complete engagement in interactions with pDNA at N/P 15. For these PAMAM–Arg conjugates, we evaluated the influence of the N/P ratio on the cytotoxicity (Figure 5B). As expected, the cytotoxicity increases with the excess of free amines introduced in the system, *i.e.*, with the increase of the N/P ratio, but remaining lower than the corresponding PAMAM G4. Nonetheless, for the best performing PAMAM G2–hfVal-Arg **1** in terms of TE, the cytotoxicity remains negligible at each N/P ratio.

Altogether, with the exception of PAMAM G4-αtfm-βAla-Arg **4** associated to low TEs and high cytotoxicity, the obtained results show the high potential of the synthesized fluorinated dendrimers as novel multifunctional non-viral gene delivery systems, with the possibility to be used for ^19^F-MRI. In particular, the most efficient PAMAM G2–hfVal-Arg **1** gathers the required characteristics for an efficient non-viral vector, such as high TE, low cytotoxicity, low manufacturing time and cost of the starting dendrimer, and easiness of functionalization.

## Conclusions

The decoration of PAMAM dendrimer non-viral vectors is an established strategy to overcome the challenges associated with their use in the gene delivery field such as (1) the high cost to produce high-generation dendrimers and (2) the TE/cytotoxicity paradox. Among the different strategies, the introduction of guanidinium groups generally facilitates the cellular uptake of the vehicle mainly due to its ability to form strong parallel hydrogen bonds with the cell surface counterparts.^30^ Another strategy that has been exploited recently relies on the decoration with fluorinated moieties to improve cellular uptake and/or decrease the cytotoxicity of the parent polymer. Herein, we propose for the first time the decoration of PAMAM dendrimers with arms containing both guanidino functional groups and one or two trifluoromethyl groups. Accordingly, we have prepared two novel fluorinated Arg-containing Michael acceptors **10–11** which, due to the presence of the highly electronegative trifluoromethyl moiety, react straightforwardly with the outer primary amines of the dendrimer without the need for any coupling reagent or catalyst (“click” functionalization). The obtained fluorinated PAMAM G2– and PAMAM G4–Arg conjugates showed from better to much better TEs than the undecorated PAMAM dendrimers and also than the gold standard bPEI 25 kDa and very low cytotoxicity at different N/P ratios. Very interestingly, the best performing conjugate resulted to be PAMAM G2–hfVal-Arg **1**, which gathers promising features for a non-viral gene vector, such as high TE, low cytotoxicity, and low cost being synthetized starting from low-generation PAMAM G2. Overall, the results presented herein all disclosed fluorinated-Arg Michael acceptors **10–12** as suitable precursors for tailoring safe and efficient multifunctional gene delivery vectors with possible implementation also in ^19^F-MRI applications.

## Experimental Procedures

### Materials

PAMAM G2 and PAMAM G4 dendrimer2 (ethylenediamine core, 16 and 64 surface groups, respectively), 25 kDa bPEI (*M*_w_ ∼ 25 kDa by LS and average *M*_n_ ∼ 10 kDa by GPC), and all other organic reactants, solvents, and culture reagents were purchased from Sigma-Aldrich (Milan, Italy) if not differently specified and used as received. The use of a cationic polymer based on PEI for transfection is covered by US Patent 6,013,240, European Patent 0,770,140, and foreign equivalents, for which Polyplus-transfection is the worldwide exclusive licensee. 2-(Trifluoromethyl)acryloyl chloride **8** was synthesized from 2-(trifluoromethyl)acrylic acid as described in the literature.^42^ Spectra/Por dialysis bags (MWCO = 1 kDa) were obtained from Spectrum Laboratories (Compton, CA, USA). The green fluorescent plasmid (Monster Green Fluorescent Protein phMGFP Vector) encoding for the modified green fluorescent protein was purchased from Promega (Milano, Italy). Cell viability was evaluated *via* automated count of the CELENA S trypan blue-stained protocol using Luna cell counting slides following the manufacturer’s instructions from Logos Biosystems. The HUVEC cell line was purchased from PromoCell (Heidelberg, Germany).

^1^H, ^13^C, and ^19^F NMR spectra were recorded on 400 MHz spectrometers. Chemical shifts are expressed in ppm (δ), using tetramethylsilane (TMS) as the internal standard for ^1^H and ^13^C nuclei (δ_H_ and δ_C_ = 0.00), while C_6_F_6_ was used as the external standard (δ_F_ −162.90) for ^19^F. ESI mass spectra were recorded by a Bruker Esquire 3000+ instrument equipped with an MS detector composed of an ESI ionization source and a single quadrupole mass selective detector. Purifications of the intermediates was performed by flash chromatography (FC) with Biotage Isolera one flash purification chromatography ISO–1SV Unit4 Pred Selekt.

### Synthesis of Methyl *N*^ω^-((2,2,4,6,7-Pentamethyl-2,3-dihydrobenzofuran-5-yl)sulfonyl)-*N*^2^-(4,4,4-trifluoro-3-(trifluoromethyl)but-2-enoyl)-l-argininate **10**

To a stirred solution of 4,4,4-trifluoro-3-(trifluoromethyl) crotonic acid **7** (113 mg, 0.54 mmol) in DCM (2.0 mL), HBTU (205 mg, 0.54 mmol) was added at 0 °C. After 10 min, a solution of H-Arg(pbf)-OMe **6** (198 mg, 0.45 mmol) in DCM (5.0 mL) was added followed by TEA (62.7 μL, 0.45 mmol). The solution was stirred at rt overnight. The solution was diluted with DCM and washed with a saturated 1 M aqueous solution of HCl, brine, a saturated aqueous solution of NaHCO_3_, and brine. The organic phase was dried over Na_2_SO_4_ and filtered, and the solvent was evaporated under pressure. The crude was purified by FC, affording 198 mg of **10** (0.32 mmol, 70% yield) as a yellowish solid.

### Synthesis of Methyl *N*^ω^-((2,2,4,6,7-Pentamethyl-2,3-dihydrobenzofuran-5-yl)sulfonyl)-*N*^2^-(2-(trifluoromethyl)acryloyl)-l-argininate **11**

To a stirred solution of H-Arg(pbf)-OMe **6** (125 mg, 0.28 mmol) in DCM (4.3 mL) at 0 °C was added DIPEA (54.4 μL, 0.33 mmol) followed by a solution of 2-(trifluoromethyl)acryloyl chloride **8** (46 mg, 0.33 mmol) in DCM (1 mL) dropwise. The solution was stirred at 0 °C for 3 h. The solution was diluted with a saturated aqueous solution of NH_4_Cl and extracted with DCM. The collected organic phases were dried over Na_2_SO_4_ and filtered, and the solvent was evaporated under pressure. The crude was purified by FC, affording 106 mg of **11** (0.19 mmol, 67% yield) as a yellowish solid.

### Synthesis of Methyl *N*^2^-Acryloyl-*N*^ω^-((2,2,4,6,7-pentamethyl-2,3-dihydrobenzofuran-5-yl)sulfonyl)-l-argininate

To a stirred solution of H-Arg(pbf)-OMe **6** (150 mg, 0.34 mmol) in DCM (5.0 mL) at 0 °C was added DIPEA (71.3 μL, 0.40 mmol) followed by a solution of acryloyl chloride **9** (33 μL, 0.40 mmol) in DCM (1 mL) dropwise. The solution was stirred at 0 °C for 3 h. The solution was diluted with a saturated aqueous solution of NH_4_Cl and extracted with DCM. The collected organic phases were dried over Na_2_SO_4_ and filtered, and the solvent was evaporated under pressure. The crude was purified by FC, affording 135 mg of **12** (0.27 mmol, 80% yield) as a white solid.

### Synthesis of PAMAM–Arg Conjugates **1–4** and **13**

#### General Procedure

To a solution of the Michael acceptors **10–12** (n*1.5 equiv., where *n* = number of the outer primary amines of the dendrimer, namely, 16 for PAMAM-G2 and 64 for PAMAM G4) in MeOH (0.5 mL), a solution of the PAMAM dendrimer (10 mg) in MeOH (0.5 mL) was added dropwise, and the solution was stirred overnight at a given temperature (40 °C for the reaction with **10**, rt for the reaction with **11**, and 70 °C for the reaction with **12**). The solvent was evaporated, the crude was dissolved in a TFA/H_2_O/TIPS (95:2.5:2.5) mixture, and the solution was stirred at rt for 3 h. The conjugates were precipitated in diethyl ether and purified *via* dialysis with a SpectraPor RC membrane (MWCO = 1 kDa) against deionized water for 2 days. The products were lyophilized, obtaining PAMAM–Arg conjugates **1–4** and **13** as fluffy white solids.

#### DLS Analysis

A double dynamic light scattering (DLS) size distribution and ζ-potential were assessed, performed on a Zetasizer Nano ZS (Malvern Instruments), considering dendrimers with and without the genetic material to evaluate the change in the dendriplex sizes. The analysis was performed in backscattering (173°) on samples prepared at the same concentrations as they were for transfections. Concentrations for transfection are reached considering 20 μL of 10 mg/mL aqueous dendrimer suspension and 5 μL of water and 4 μL of 1 μg/μL DNA mixed with 1 μL of 100 mM NaOAc separately. Then, a portion of dendritic solution was mixed into 5 μL of the aqueous phase containing the genetic material at a concentration that allowed us to establish the desired N/P ratio, and the dendriplex suspension was left to complex for about 10 min at room temperature and properly diluted in PBS to reach 0.6 μg DNA dose. For DLS analysis, concentrations used were the same for transfection, but dendriplex solutions were scaled up to a final volume of 1.6 mL for a proper analysis.

#### Agarose Gel Retardation Assay

Dendrimers/DNA complexes at different N/P ratios (5, 15, and 30) were prepared following complexation instructions of the transfection protocol. 250 ng of pGFP or model DNA (herring sperm for proof-of-concept assays), both from Promega, was used to form complexes with a proper dilution of the dendrimer in deionized water. Complexes were then diluted to 20 μL final volume, and 4 μL of loading dye (blue/orange loading dye, 6× from Promega) was added to dendriplexes. 24 μL of each sample was loaded on 0.8% (w/v) agarose gel. DNA ladder (BenchTop 1 kb DNA Ladder, from Promega) was loaded in the first lane, while free DNA was loaded in the last one. Electrophoresis running was performed in Tris-acetate-EDTA buffer (1× TAE) at 150 V for 30 min. The gel was subsequently placed into a plastic tray and incubated with staining solution (Diamond Nucleic Acid Dye Promega diluted in 1× TAE buffer) following the manufacturer’s instructions, for 20 min under gentle shaking, protected from light. The gel was analyzed under UV Transilluminator BIO-RAD ChemiDoc XRS+ using a proper filter.

#### Transfection and Viability Protocols

Following a specific transfection protocol, biological tests in vitro were performed. In this work, the green fluorescent protein plasmid (pGFP) and HUVECs were used for transfection efficiency and cytotoxicity evaluation of the four PAMAM–Arg conjugates synthesized. Transfection tests were performed preparing 20 μL of 10 mg/mL aqueous dendrimer suspension and 5 μL of water and 4 μL of 1 μg/μL DNA mixed with 1 μL of 100 mM NaOAc separately. Then, a portion of dendritic solution was mixed into 5 μL of the aqueous phase containing the genetic material at a concentration that allowed us to establish the desired N/P ratio, and the dendriplex suspension was left to complex for about 10 min at room temperature. The dendriplexes were properly diluted at first in PBS and then with the culture medium (endothelial cell growth medium MV from PromoCell) to reach 0.6 μg DNA dose. 150 μL of media/dendriplex solution was added per well for 15′000 cells per well in a 96-well plate. For cytotoxicity tests without the plasmid cargo, the dendrimer suspension was mixed with 5 μL of PBS. Cell viability was evaluated *via* automated count of the CELENA S trypan blue-stained protocol using Luna Cell Counting Slides following the manufacturer’s instructions.

#### Statistical Analysis

Student’s *t*-test was used to determine statistical significance. For each analysis, for test *in vitro*, almost 4 replicates for each polymer were done and almost 10 images for each replicate were acquired with CELENA S using PlanAchro 4× Objective lens, avoiding a prolongate light microscope exposure of culture cells.

#### Image Analysis for GFP Fluorescence and Percentage of Transfected Cells

For transfection efficiency studies, the GFP fluorescence intensity measure and the percentage of transfected cells were evaluated with ImageJ.^48,49^ Cell counting has been settled with the software analyzing for each image with specific tools such as brightness and contrast, thresholding method, and find edges to perform the cell count implemented with the Analyze Particle tool. The final percentage of transfected cells was obtained considering the ratio between the number of transfected cells and the total number of cells of the images acquired with CELENA S with and without a GFP filter cube, respectively. GFP fluorescence intensity was estimated with ImageJ software through the corrected total cell fluorescence (CTCF) measure

CTCF = integrated density – (area of selected cell × mean fluorescence of background readings).^50,51^

## Supporting Information Available

Experimental procedures and chemical characterizations of model compounds **15–17**; ^1^H, ^13^C, and ^19^F NMR spectra of all new compounds; agarose gel analysis of the model DNA and pGFP used for preliminary complexation behavior analysis for PAMAM G4−αtfm-βAla-Arg **4** and PAMAM G2 at different N/P ratios; and TEs of each test normalized by the TE of the positive control bPEI 25 kDa (PDF)
