# Supplementary material for: Selectively Fluorinated PAMAM–Arginine Conjugates as Gene Delivery Vectors
Source: Bioconjug Chem. 2023 May 23;34(6):1084–95. doi: 10.1021/acs.bioconjchem.3c00139 (PMC10288444; doi:10.1021/acs.bioconjchem.3c00139)

SUPPORTING INFORMATION

Selectively Fluorinated PAMAM-Arginine Conjugates as Gene Delivery Vectors

Carola Romani,[§] Paola Gagni,[†] Mattia Sponchioni,[§,*] Alessandro Volonterio[§,†,*]

Department of Chemistry, Materials and Chemical Engineering "Giulio Natta", Politecnico di Milano, via Mancinelli 7, 20131 Milano, Italy; Consiglio Nazionale delle Ricerche, Istituto di Scienze e Tecnologie Chimiche "Giulio Natta" (SCITEC), Via Mario Bianco 9, 20131 Milan, Italy.

Table of contents

Pages S2 Scheme S1. Synthesis of model compounds **15-17** and experimental procedure

Page S3-S4 Chemical characterization of all new compounds.

Pages S5-S15 Copies of the ^{1}H NMR, ^{19}F NMR, ^{13}C NMR and ESI MS spectra of all new compounds

Pages S16-S20 Copies of the ^{1}H NMR and ^{19}F NMR of PAMAM-Arg conjugates **1-4, 13**

Page S21 Figure S1. Agarose gel analysis of the model DNA (S1a) and pGFP (S1b) used as preliminary complexation behavior analysis for PAMAM G4-αtfm-βAla-Arg **4** and PAMAM G2 at different N/P ratios.

Page S22 Figure S2 Particle size distribution by intensity, volume, and number for the four dendriplexes synthesized

Page S23 Figure S3. TEs of each tested systems (considering N/P ratio equal to 15) normalized by the TE of the positive control bPEI 25kDa

Scheme S1. Synthesis of model compounds **15-17**

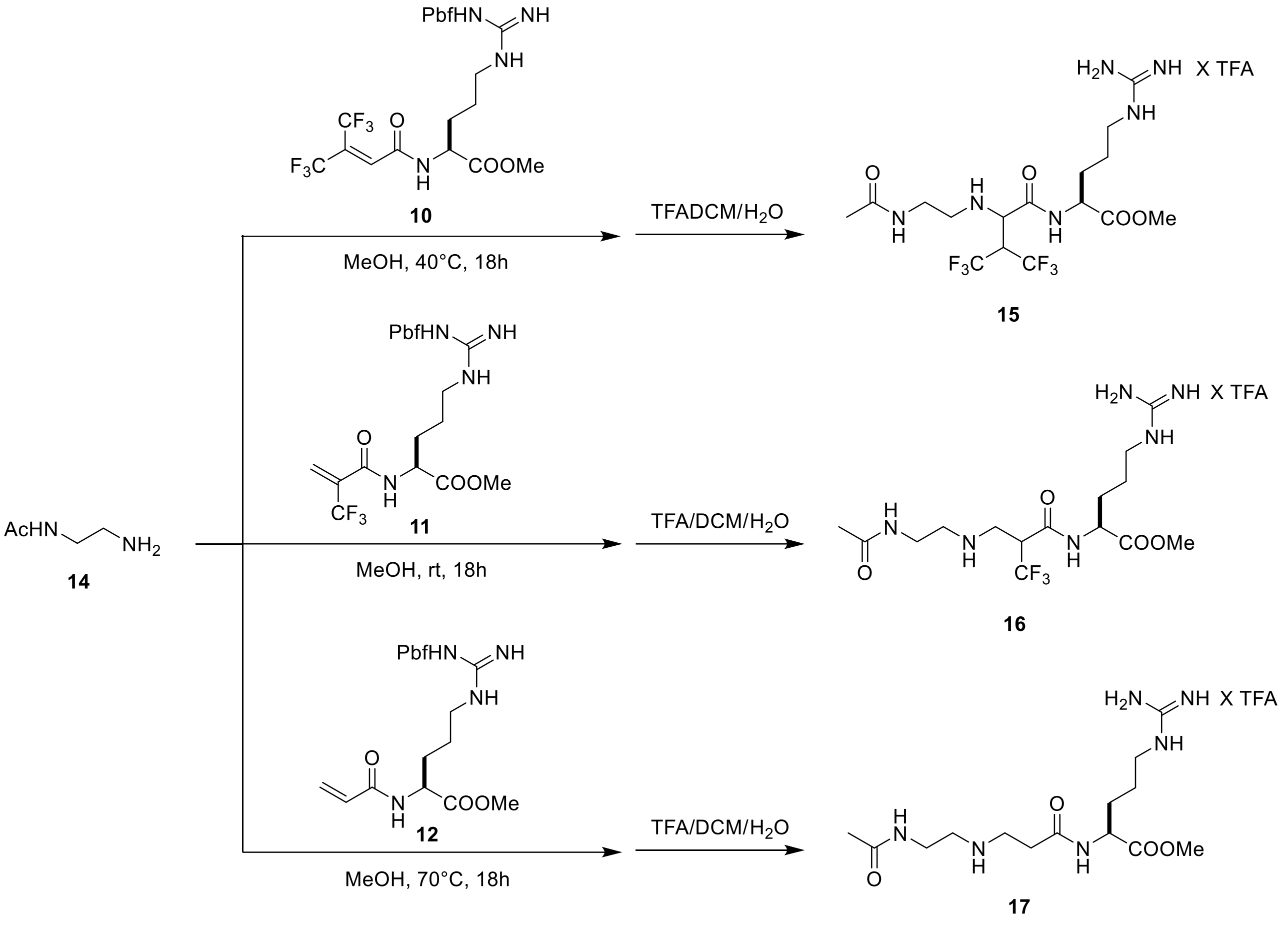

General procedure. To a solution of the Michael acceptors **10-12** (1.0 equiv.) in MeOH (0.1 M solution) a solution of N-(2-aminoethyl)acetamide **14** (1.2 equiv.) in MeOH (0.5 M solution) was added dropwise and the solution stirred overnight at a given temperature (40 °C for the reaction with **10**, rt with **11**, and 70°C with **12**). The solvent was evaporated, the crude dissolved in AcOEt and washed with an aqueous 1M solution of HCl and brine. The organic layer was separated, dried over Na_2SO_4, filtered and the solvent removed under pressure. The crude dissolved in a DCM/TFA/H_2O (25:70:5) mixture and the mixture stirred at rt for three hours. The DCM was evaporated and compounds **15-17** were precipitate in diethyl ether.

methyl N$^{\omega}$-((2,2,4,6,7-pentamethyl-2,3-dihydrobenzofuran-5-yl)sulfonyl)-N2-(4,4,4-trifluoro-3-(trifluoromethyl)but-2-enoyl)-L-argininate, 10. ^{1}H NMR (400 MHz, $CDCl_3$) δ 7.62 (d, *J* = 8.0 Hz, 1H), 7.08 (s, 1H), 6.30-6.28 (br s, 3H), 4.56-4.51 (m, 1H), 3.69 (s, 3H), 3.20-3.18 (m, 2H), 2.94 (s, 2H), 3.31 (s, 3H), 3.29 (s, 3H), 2.07 (s, 3H), 1.88-1.86 (m, 1H), 1.81-1.79 (m, 1H), 1.60.1,57 (m, 2H), 1.45 (s, 6H); ^{19}F NMR ($CDCl_3$, 376 MHz) δ – 62.1 (q, *J* = 6.8 Hz, 3F), 67.0 (q, *J* = 6.8 Hz, 3F); ^{13}C NMR (101 MHz, $CDCl_3$) δ 169.7, 169.4, 160.0, 157.1, 154.3, 136.4, 134.0, 130.3, 122.9, 117.9 (q, *J* = 276.7), 115.8, 84.7, 58.5, 50.6, 50.5, 41.2, 38.7, 27.0, 26.6, 23.3, 19.1, 17.2, 15.8, 12.2, 10.4, the carbon bonding the two CF_3 groups did not appear due to low intensity; ESI m/z 631.3 [M+H, (100)]$^+$; Anal. calcd. for $C_{25}H_{32}F_6N_4O_6S$: C 47.62, H 5.12, N, 8.88; found: C 47.63, H 5.11, N, 8.88.

methyl N$^{\omega}$-((2,2,4,6,7-pentamethyl-2,3-dihydrobenzofuran-5-yl)sulfonyl)-N^{2}-(2-(trifluoromethyl)acryloyl)-L-argininate, 11. ^{1}H NMR (400 MHz, $CDCl_3$) δ 7.25 (d, *J* = 7.2 Hz, 1H), 6.43 (s, 1H), 6.34 (s, 1H), 6.28 (br s, 2H), 6.16 (s, 1H), 4.56 (br q, *J* = 4.8 Hz, 1H), 3.72 (s, 3H), 3.22-3.20 (m, 2H), 2.97 (s, 2H), 2.55 (s, 3H), 2.48 (s, 3H), 2.10 (s, 3H), 1.96-1.94 (m, 1H), 1.93-1.91 (m, 1H), 1.63-1.61 (m, 2H), 1.46 (s, 6H); ^{19}F NMR ($CDCl_3$, 376 MHz) δ – 64.5 (s, 3F); ^{13}C NMR (101 MHz, $CDCl_3$) δ 172.1, 162.0, 158.9, 156.4, 138.2, 133.4 (d, *J* = 31.3 Hz), 132.6, 132.1, 128.6, 124.8, 121.8 (q, *J* = 274.7 Hz), 117.6, 86.5, 60.5, 52.6, 43.2, 40.5, 29.1, 28.6, 25.4, 19.2, 17.9, 14.2, 12.4; ESI m/z 563.3 [M+H, (100)]$^+$; ESI m/z 585.3 [M+Na, (20)]$^+$; Anal. calcd. for $C_{24}H_{33}F_3N_4O_6S$: C 51.24, H 5.91, N 9.96; found: C 51.22, H 5.90, N 9.97.

methyl N^{2}-acryloyl-N$^{\omega}$-((2,2,4,6,7-pentamethyl-2,3-dihydrobenzofuran-5-yl)sulfonyl)-L-argininate, 12. ^{1}H NMR (400 MHz, $CDCl_3$) δ 7.02 (d, *J* = 7.6 Hz, 1H), 6.48 (br s, 3H), 6.26 (br s, 1H), 6.25 (m, 1H), 5.65 (dd, *J* = 8.8 and 2.8 Hz, 1H), 4.58 (br q, *J* = 4.8 Hz, 1H), 3.73 (s, 3H), 2.25-2.22 (m, 2H), 2.97 (s, 2H), 2.57 (s, 3H), 2.51 (s, 3H), 2.11 (s, 3H), 1.90-1.86 (m, 1H), 1.81-1.78 (m, 1H), 1.65-1.61 (m, 2H), 1.48 (s, 3H), 1.47 (s, 3H); ^{13}C NMR (101 MHz, $CDCl_3$) δ 172.5, 166.1, 159.1, 156.0, 138.6, 132.5, 130.2, 127.5, 124.8, 117.7, 86.6, 52.5, 43.2, 40.7, 29.6, 28.6, 25.3, 19.3, 17.9, 12.5; ESI m/z 517.3 [M+Na, (100)]$^+$; Anal. calcd. for $C_{23}H_{34}N_4O_6S$: C 55.85, H 6.93, N 11.33; found: C 55.87, H 6.93, N 11.34.

methyl (2-((2-acetamidoethyl)amino)-4,4,4-trifluoro-3-(trifluoromethyl)butanoyl)-L-argininate, 15. ^{1}H NMR (D_2O, 400 MHz), mixture of two diastereoisomers: δ 4.47-4.46 (m, 1H), 4.44 (br s, 1H), 4.34-4.32 (m, 1H), 3.74 (s, 1,5H, one diast.) 3.73 (s, 1,5H, one diast.), 3.48-3.46 (m, 1H), 3.41-3.39 (m, 1H), 3.16-3.14 (m, 2H), 3.13-2.98 (m, 2H), 1.98 (s, 3H), 1.96-1.94 (m, 1H), 1.82-1.79 (m, 1H), 1.61-1.58 (m, 2H). ^{19}F NMR (D_2O, 376 MHz), mixture of two diastereoisomers: δ – 62.6 (quintet, *J* = 9.8

Hz, 3F one diast.), – 62.9 (br s, 6F, one diast.), – 63.3 (quintet, *J* = 9.8 Hz, 3F one dist.); ESI m/z 481.2 [M+H, (100)]$^+$; Anal. calcd. for $C_{16}H_{26}F_6N_6O_4$: C 40.00, H 5.46, N, 17.49; found: C 39.98, H 5.46, N, 17.48.

methyl (2-(((2-acetamidoethyl)amino)methyl)-3,3,3-trifluoropropanoyl)-L-argininate, 16. ^{1}H NMR (D_2O, 400 MHz), mixture of two diastereoisomers: δ 4.59 (dd, *J* = 9.2 and 4.4 Hz, 0.5H, one diast.), 4.17 (dd, *J* = 8.4 and 4.8 Hz, 0.5H, one dist.), 3.95-3.87 (m, 1H), 3.76 (s, 1.5H, one dist.), 3.74 (s, 1.5H, one diast.), 3.70-3.65 (m, 1H), 3.62-3.57 (m, 1H), 3.54-3.16 (m, 2H), 3.27-3.24 (m, 2H), 3.20-3.17 (m, 2H), 1.99 (s, 3H), 1.98-1.94 (m, 1H), 1.83-1.78 (m, 1H), 1.68-1.61 (m, 2H). ^{19}F NMR (D_2O, 376 MHz), mixture of two diastereoisomers: δ – 66.9 (d, *J* = 7.5 Hz, 3F one diast.), – 67.1 (d, *J* = 7.5 Hz, 3F one dist.); ESI m/z 413.1 [M+H, (100)]$^+$; Anal. calcd. for $C_{15}H_{27}F_3N_6O_4$: C 43.69, H 6.60, N 20.38; found: C 43.70, H 6.61, N 20.40.

methyl (3-((2-acetamidoethyl)amino)propanoyl)-L-argininate, 17. ^{1}H NMR (D_2O, 400 MHz): δ 4.41 (dd, *J* = 8.8 and 5.2 Hz, 1H), 3.50 (br t, *J* = 5.6 Hz, 2H), 3.47 (s, 3H), 3.32 (br t, *J* = 7.2 Hz, 2H), 3.10-3.16 (m, 4H), 2.77 (t, *J* = 6.4 Hz, 2H), 1.99 (s, 3H), 1.90-1.88 (m, 1H), 1.77-1.75 (m, 1H), 1.65-1.63 (m, 2H) ; ESI m/z 345.1 [M+H, (100)]$^+$; Anal. calcd. for $C_{14}H_{28}N_6O_4$: C 48.82, H 8.19, N 24.40; found: C 48.82, H 8.20, N 24.41.

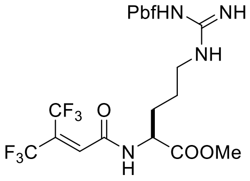
PbfHN
NH
NH
CF3
O
F3C
N
H
COOMe

10

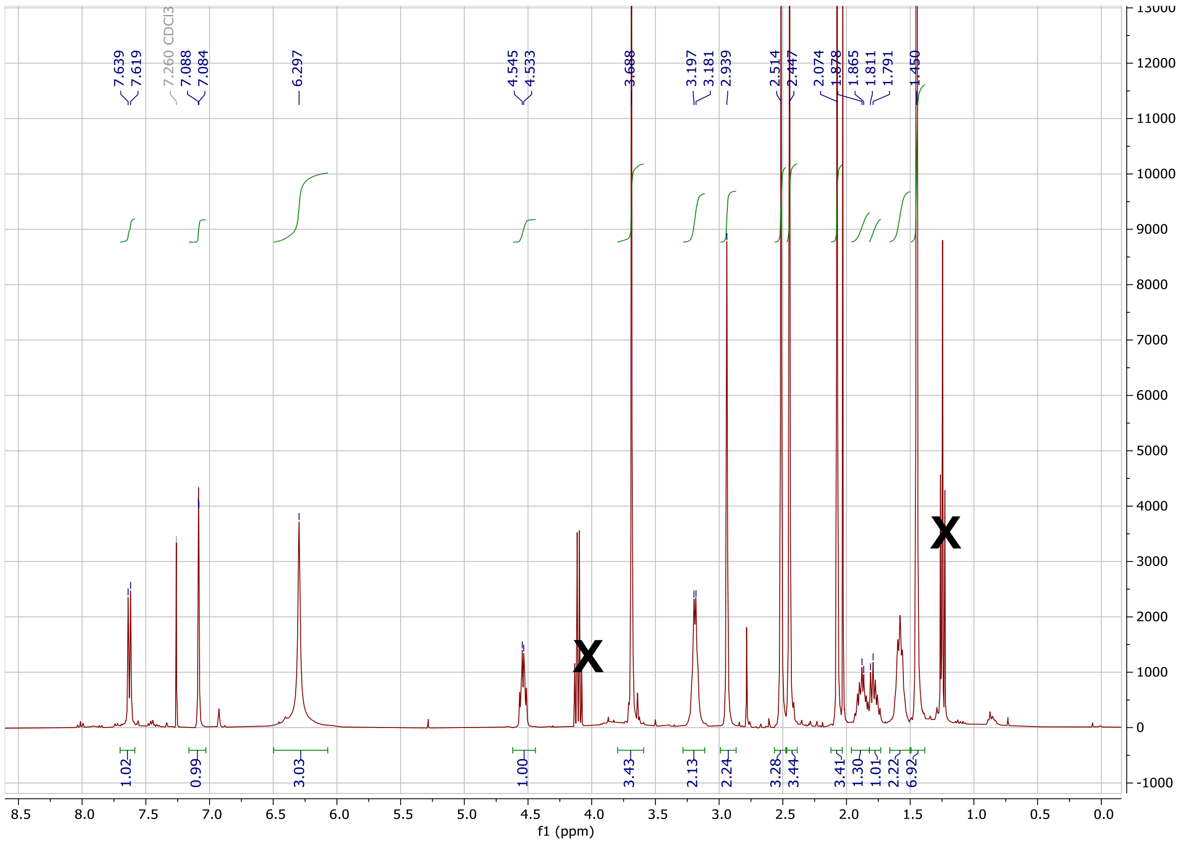
7.639
7.619
7.260 CDCl3
7.088
7.084
6.297
4.545
4.533
3.688
3.197
3.181
2.939
2.514
2.447
2.074
1.878
1.865
1.811
1.791
1.450
1.02
0.99
3.03
1.00
3.43
2.13
2.24
3.28
3.44
3.41
1.30
1.01
2.22
6.92
X
X
f1 (ppm)

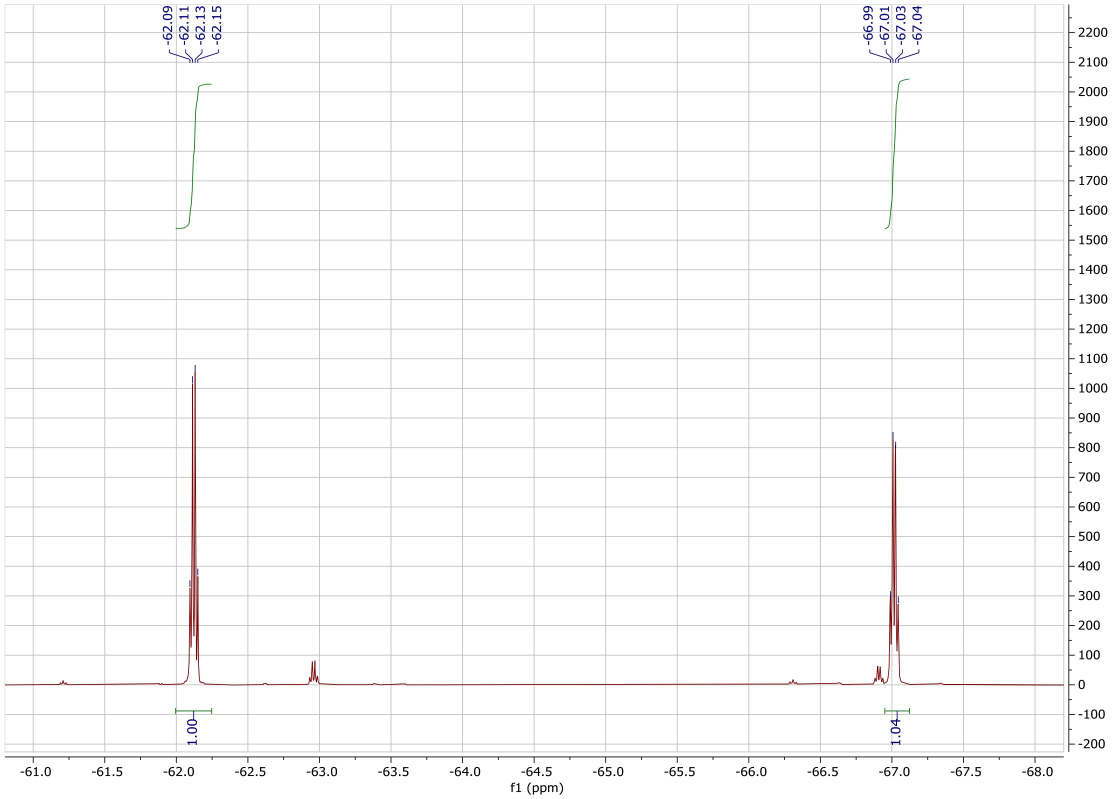
-62.09
-62.11
-62.13
-62.15
-66.99
-67.01
-67.03
-67.04
1.00
1.04
f1 (ppm)

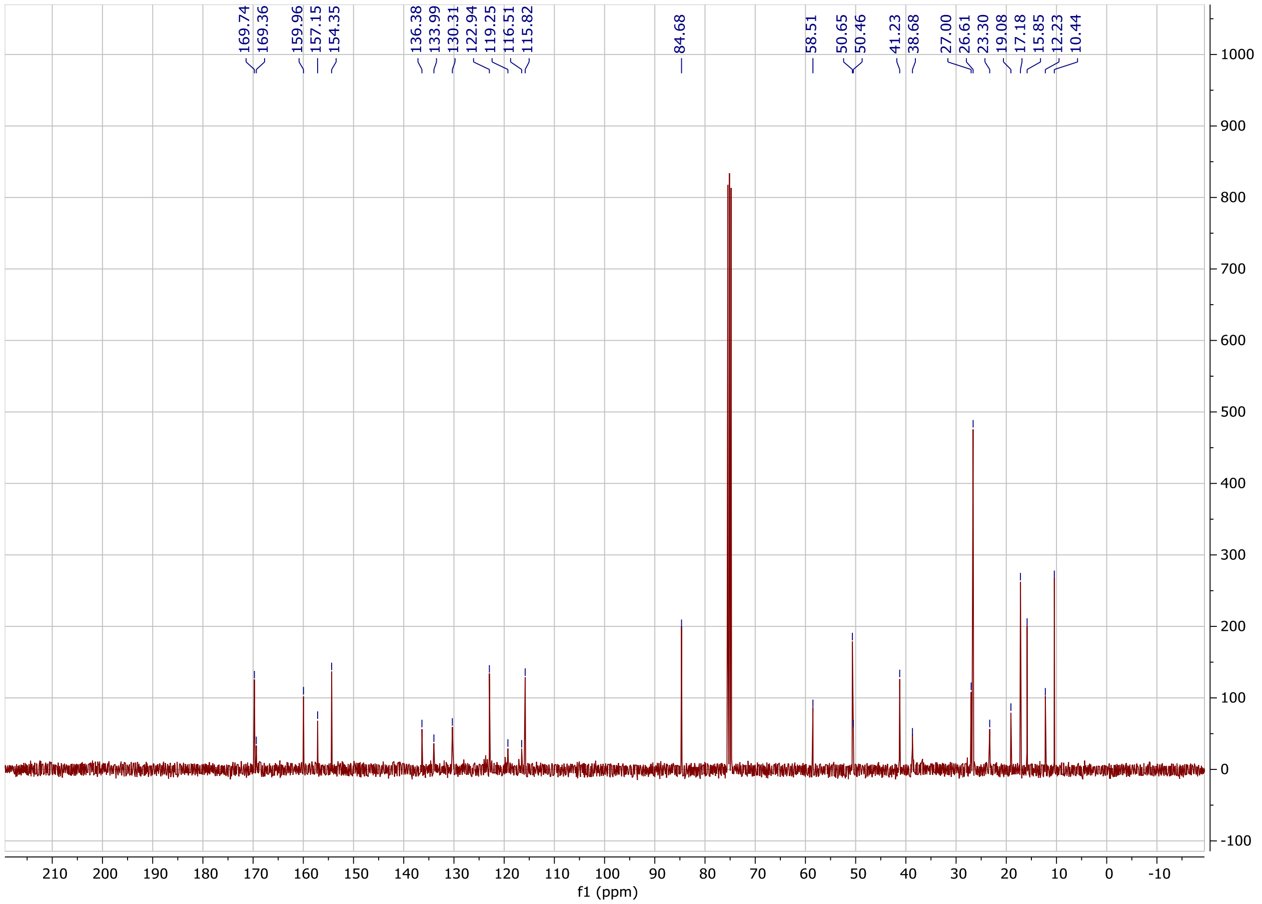

-L.G.S. - Laboratorio Grandi Strumenti - Display Report

Analysis Name	av cr74.d	Acquisition Date	10/12/21 15:19:36	Operator	Walter Panzeri
Sample Name		Method	Copy of $wp_lm.MS	Instrument	esquire3000plus
Comment	1 mg/mL dil 1:100 MeOH<br>Richiedente: Romani				

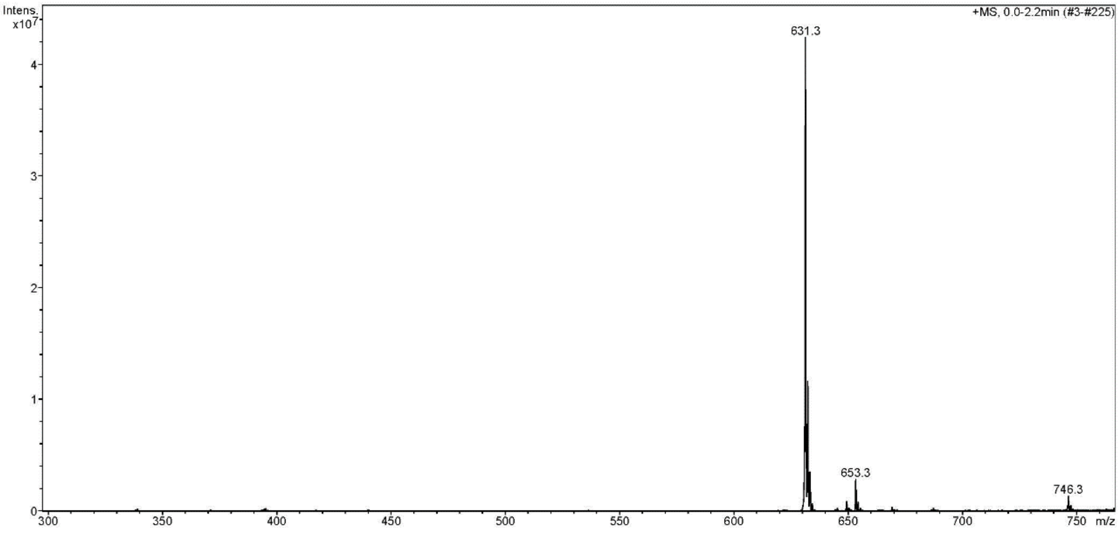

Bruker Daltonics DataAnalysis 3.1 printed: 10/12/21 15:23:05 Page 1 of 1

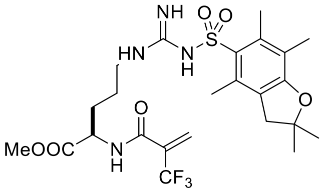
NH
O O
HN
N
S
H
O
O
MeOOC
N
H
CF3

11

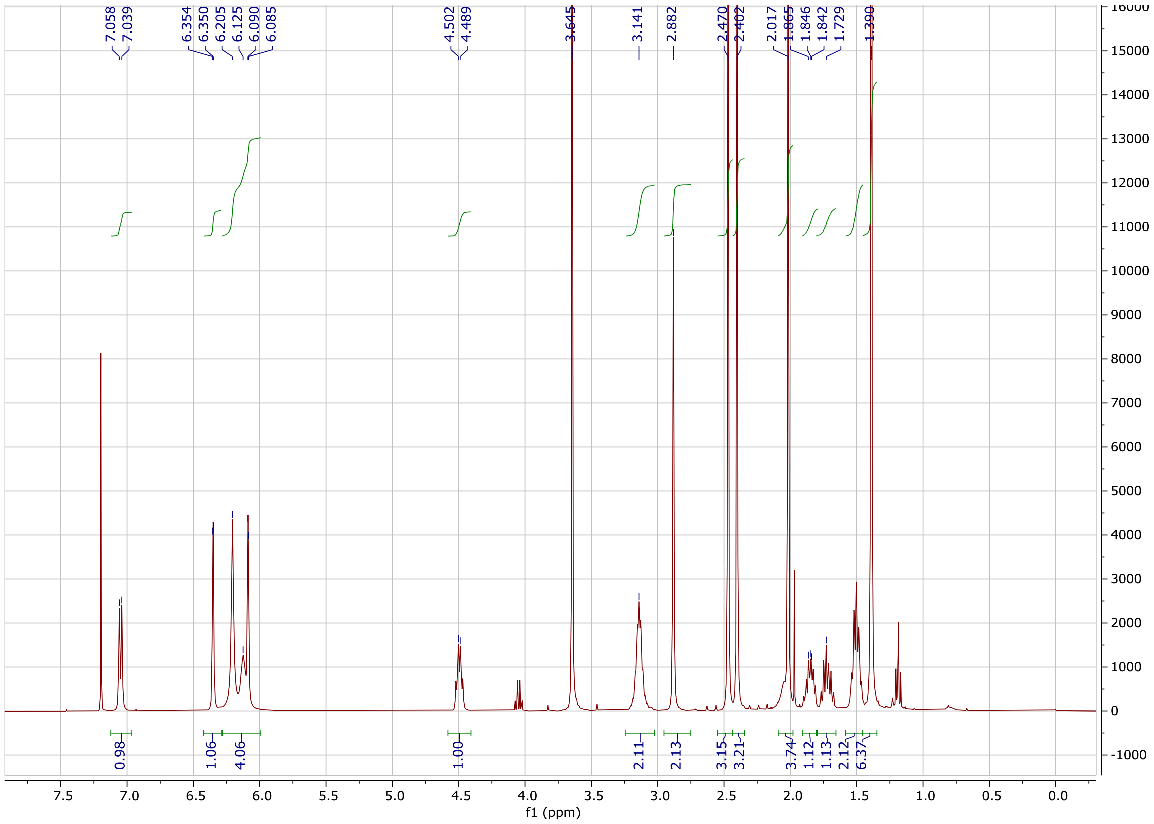
7.058
7.039
6.354
6.350
6.205
6.125
6.090
6.085
4.502
4.489
3.645
3.141
2.882
2.470
2.402
2.017
1.865
1.846
1.842
1.729
1.390
0.98
1.06
4.06
1.00
2.11
2.13
3.15
3.21
3.74
1.12
1.13
2.12
6.37
f1 (ppm)

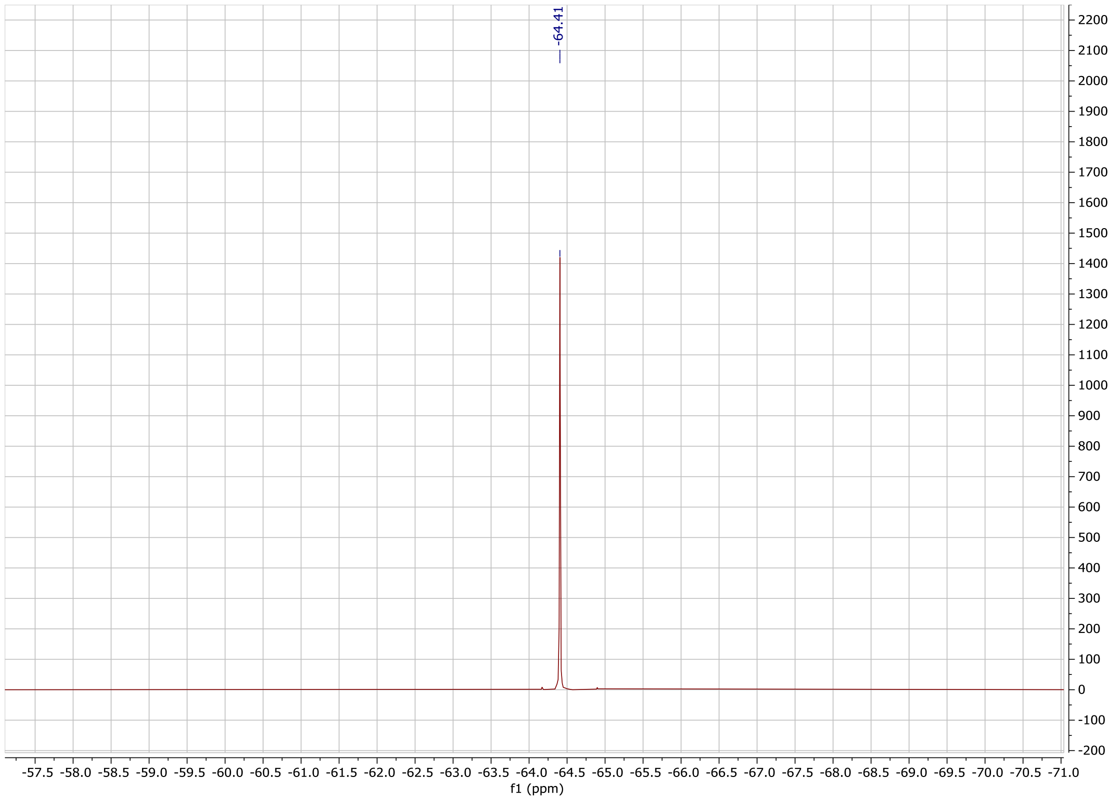
-64.41
f1 (ppm)

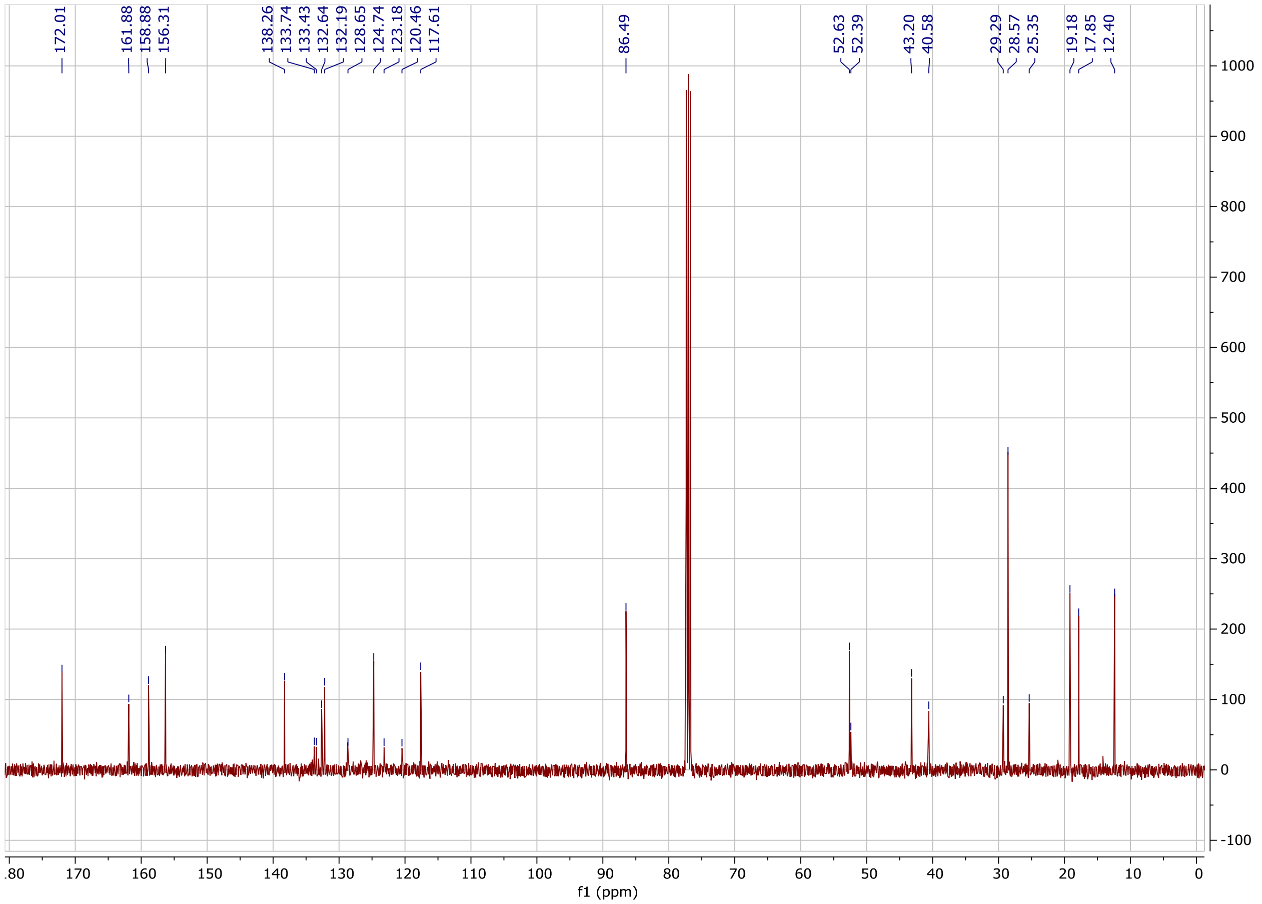

-L.G.S. - Laboratorio Grandi Strumenti - Display Report

Analysis Name	av cr75_cv10.d	Acquisition Date	09/09/21 08:46:01	Operator	Walter Panzeri
Sample Name		Method	Copy of $wp_lm.MS	Instrument	esquire3000plus
Comment	1 mg/mL dil 1:100 CH3CN<br>Richiedente: Romani				

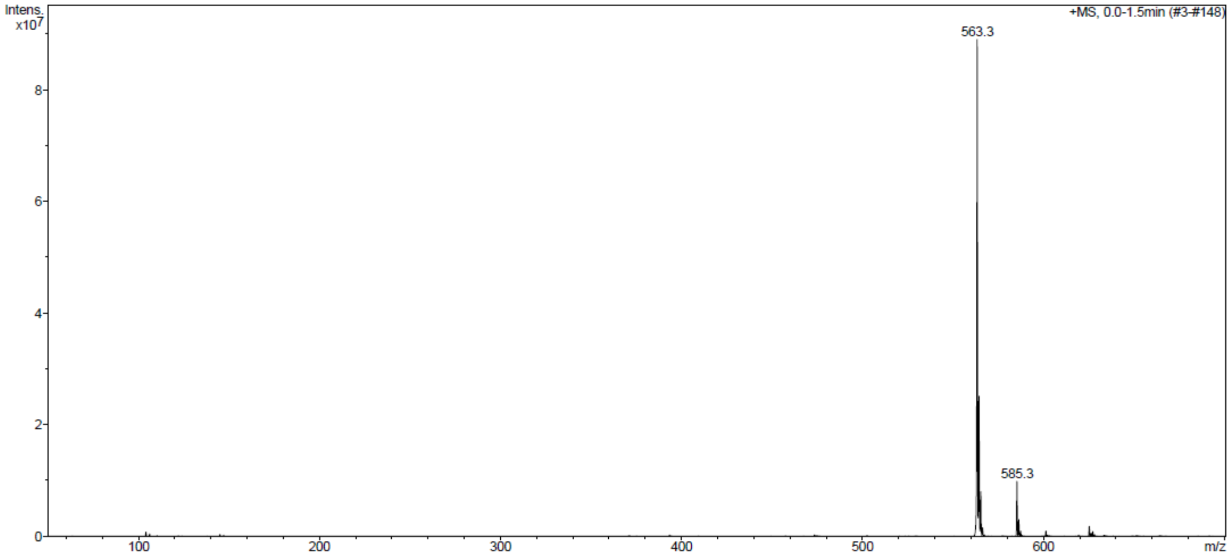

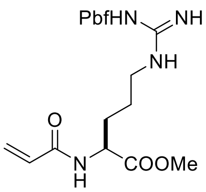
PbfHN
NH
NH
O
N
H
COOMe

12

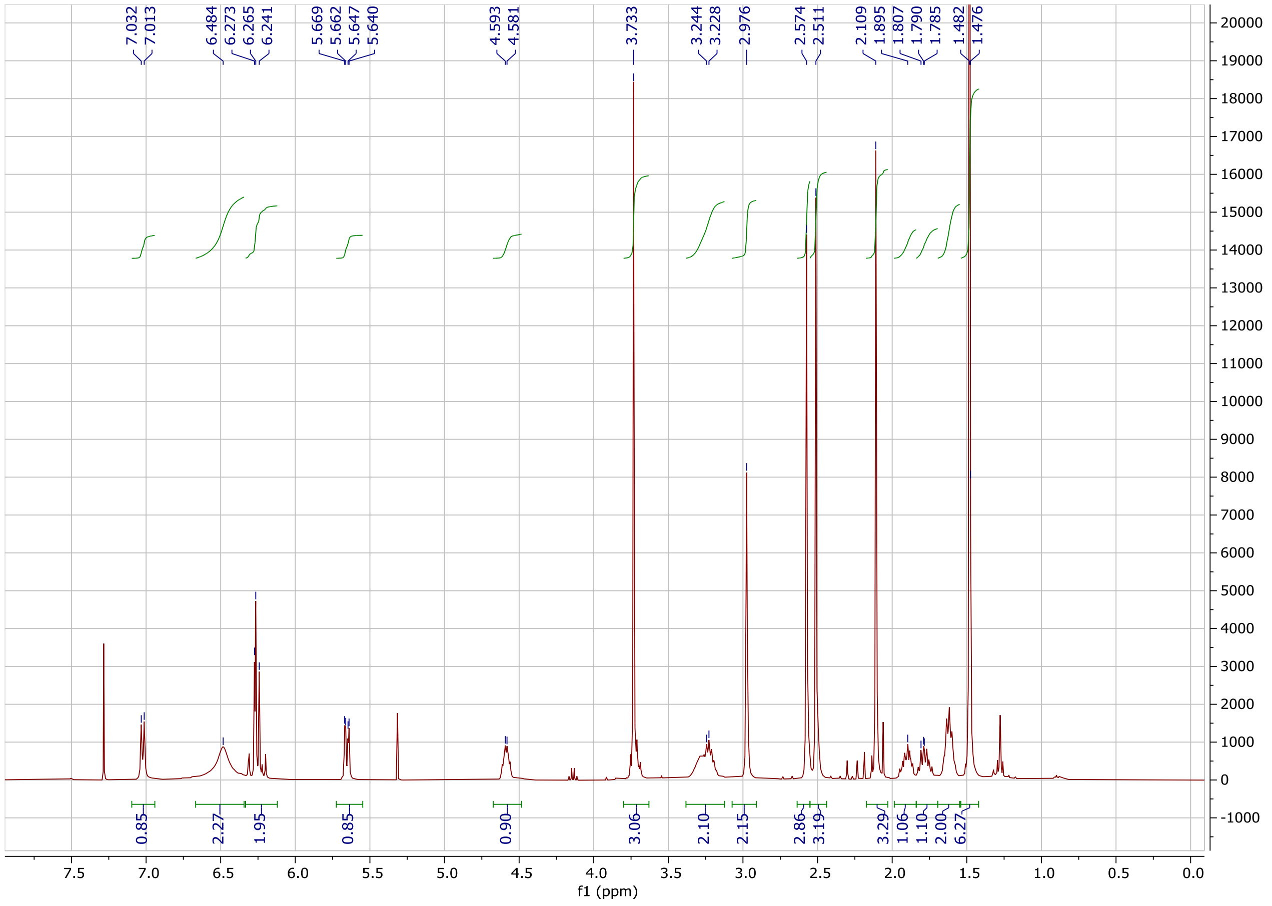
7.032
7.013
6.484
6.273
6.265
6.241
5.669
5.662
5.647
5.640
4.593
4.581
3.733
3.244
3.228
2.976
2.574
2.511
2.109
1.895
1.807
1.790
1.785
1.482
1.476
0.85
2.27
1.95
0.85
0.90
3.06
2.10
2.15
2.86
3.19
3.29
1.06
1.10
2.00
6.27
f1 (ppm)

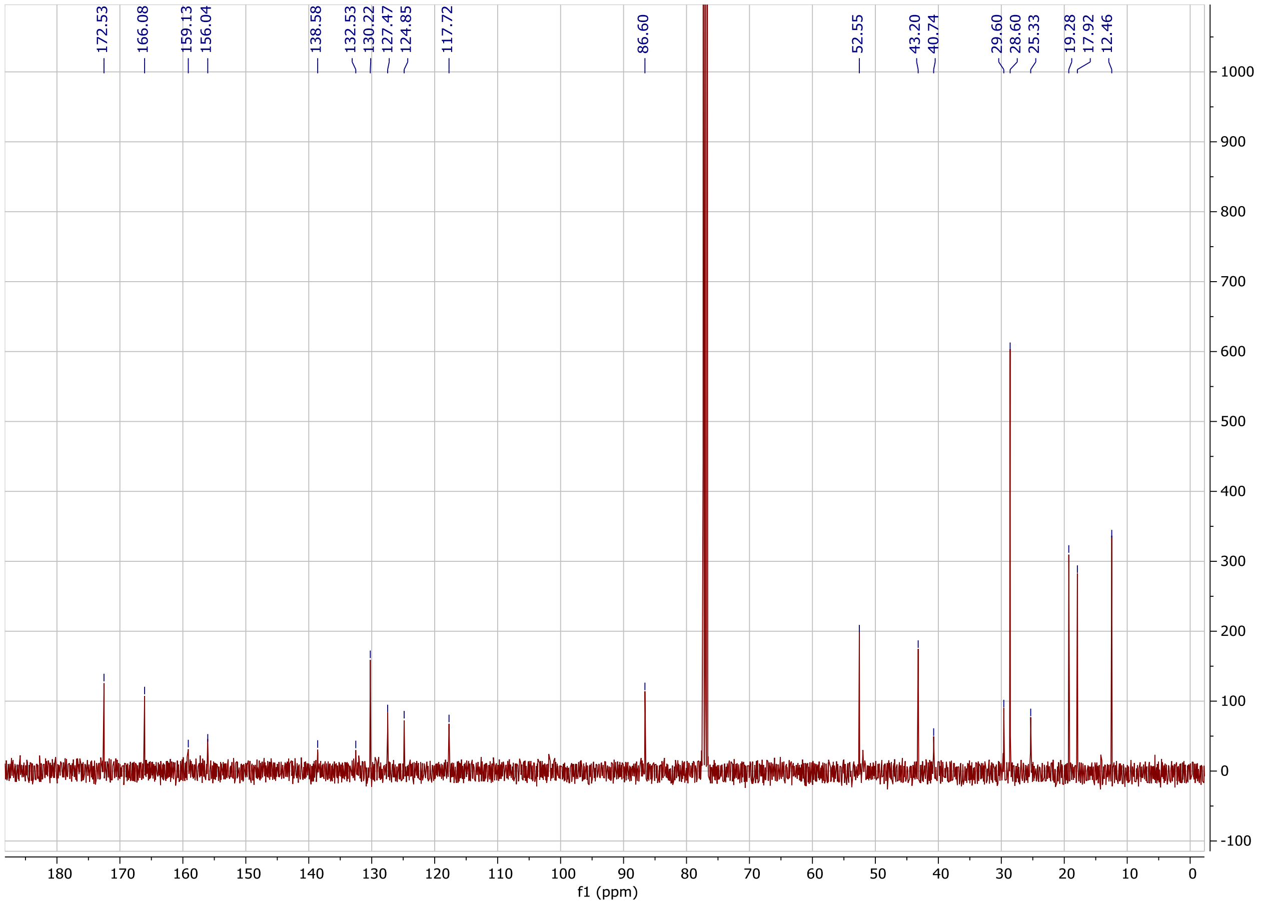

172.53
166.08
159.13
156.04
138.58
132.53
130.22
127.47
124.85
117.72
86.60
52.55
43.20
40.74
29.60
28.60
25.33
19.28
17.92
12.46
f1 (ppm)

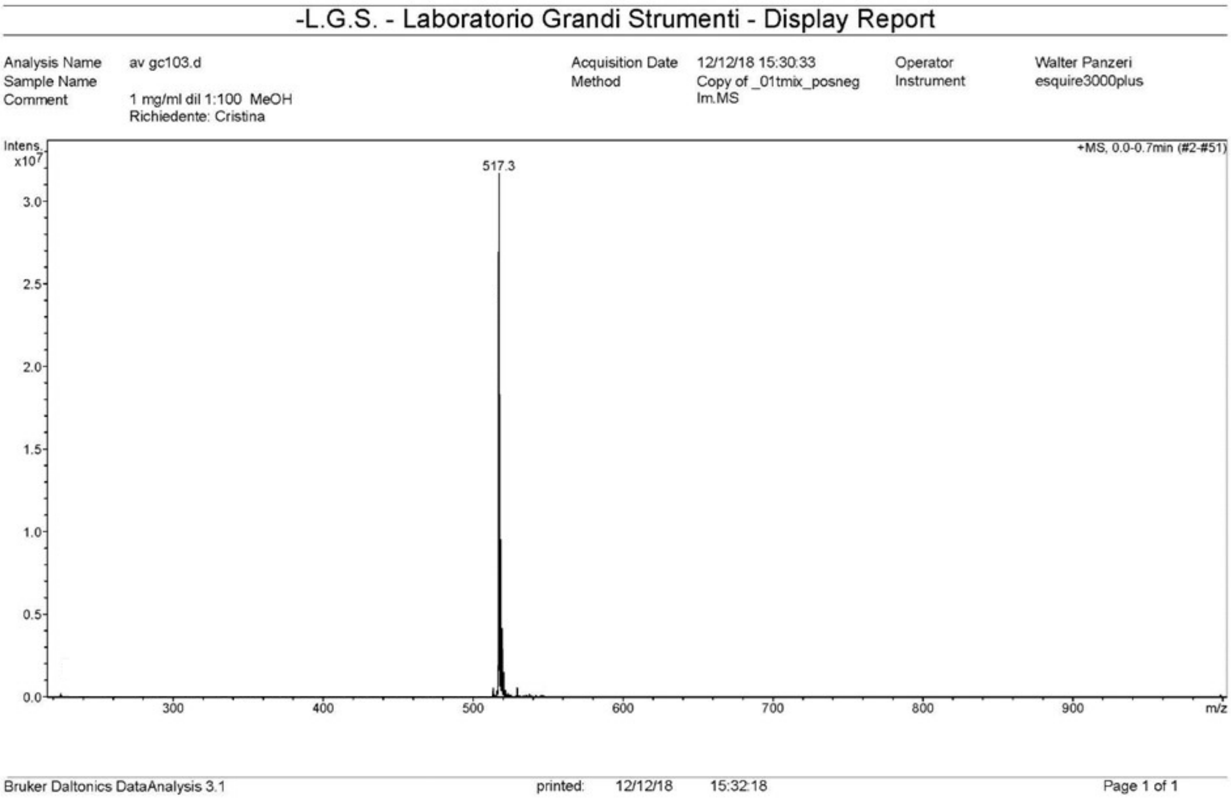

-L.G.S. - Laboratorio Grandi Strumenti - Display Report
Analysis Name av gc103.d
Sample Name
Comment 1 mg/ml dil 1:100 MeOH
Richiedente: Cristina
Acquisition Date 12/12/18 15:30:33
Method Copy of _01tmix_posneg lm.MS
Operator Walter Panzeri
Instrument esquire3000plus
Intens. x10^7
+MS, 0.0-0.7min (#2-#51)
517.3
m/z
Bruker Daltonics DataAnalysis 3.1
printed: 12/12/18 15:32:18
Page 1 of 1

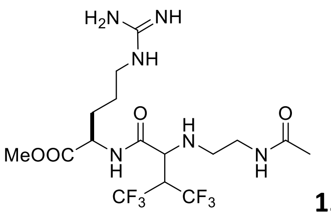
H2N
NH
NH
O
H
N
O
MeOOC
N
H
N
H
CF3
CF3

15

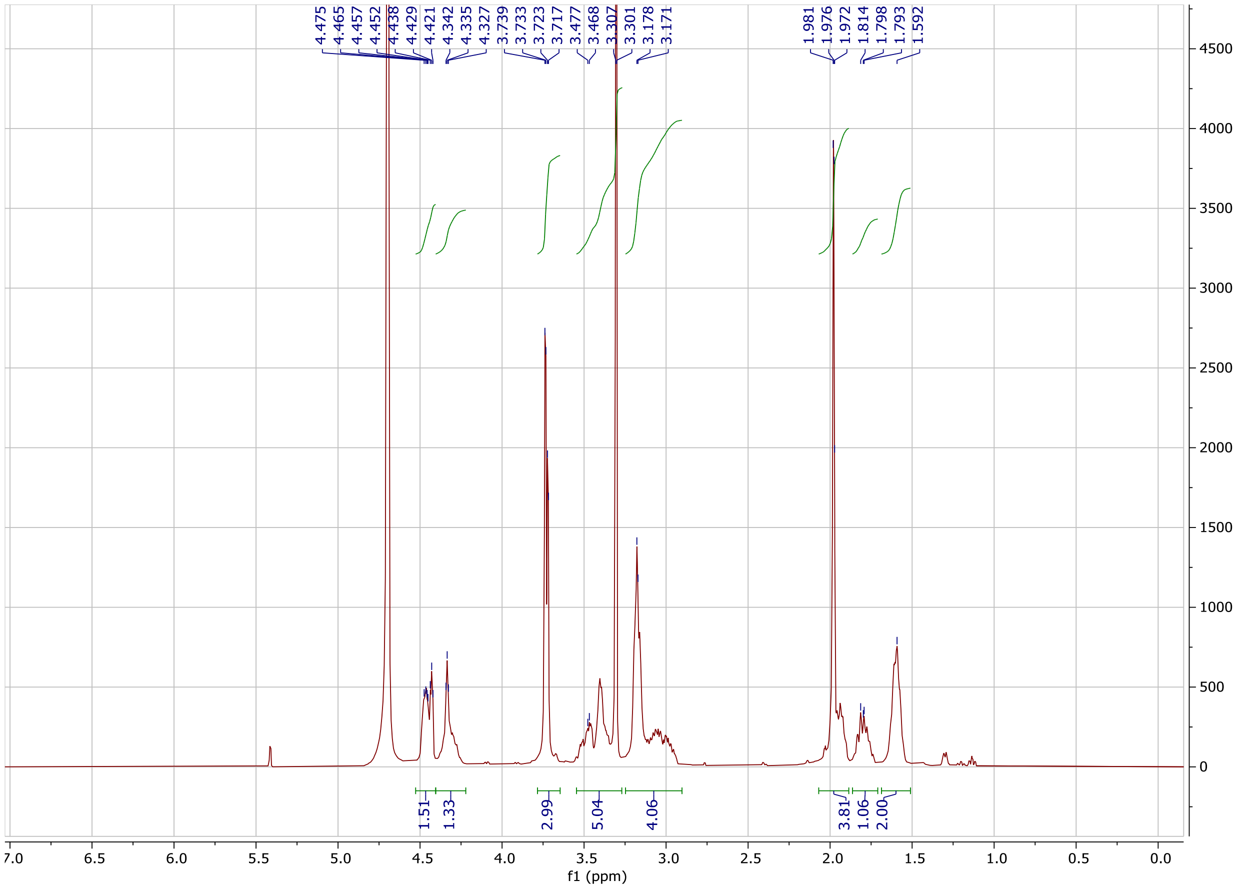
4.475
4.465
4.457
4.452
4.438
4.429
4.421
4.342
4.335
4.327
3.739
3.733
3.723
3.717
3.477
3.468
3.307
3.301
3.178
3.171
1.981
1.976
1.972
1.814
1.798
1.793
1.592
1.51
1.33
2.99
5.04
4.06
3.81
1.06
2.00
f1 (ppm)

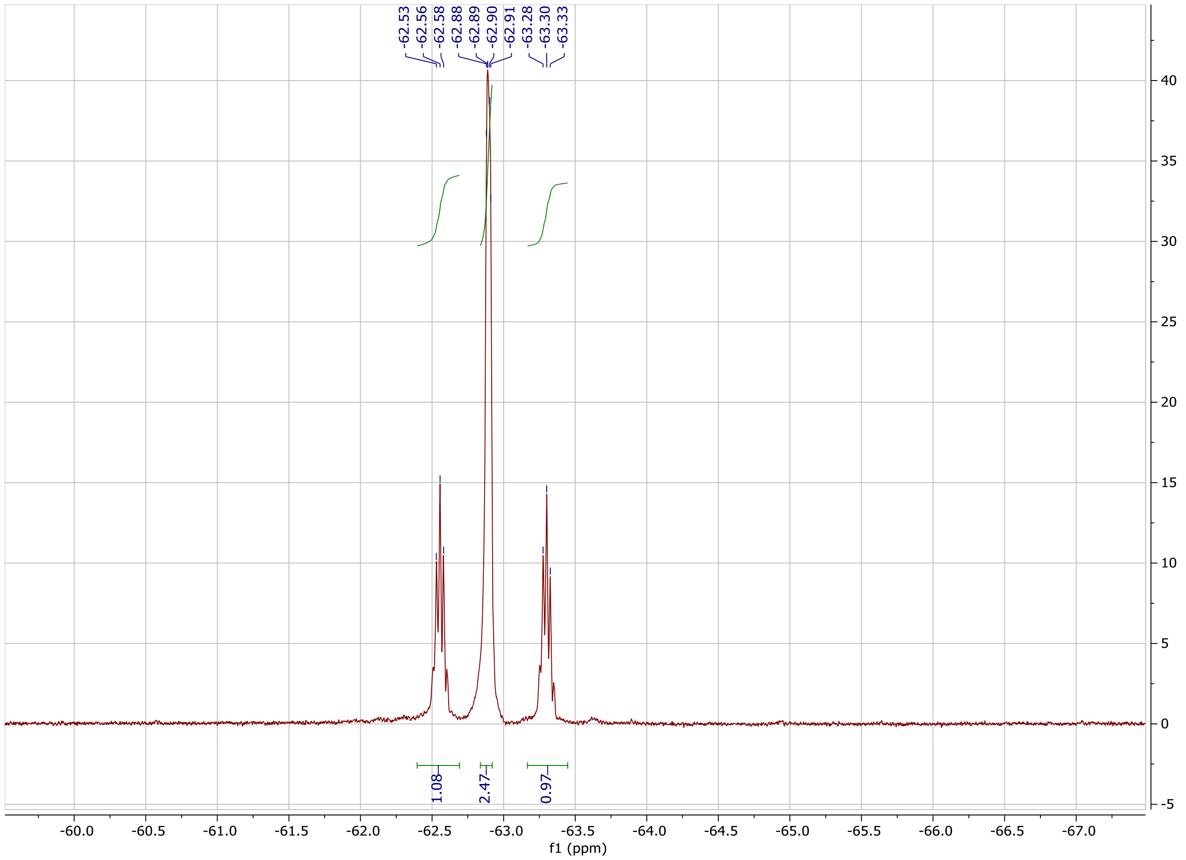

-L.G.S. - Laboratorio Grandi Strumenti - Display Report

Analysis Name	av elp134.d	Acquisition Date	09/27/19 10:25:54	Operator	Walter Panzeri
Sample Name		Method	Copy of _01tmix_posneg lm.MS	Instrument	esquire3000plus
Comment	1 mg/mL dil 1:100 MeOH<br>Richiedente: Lopresti				

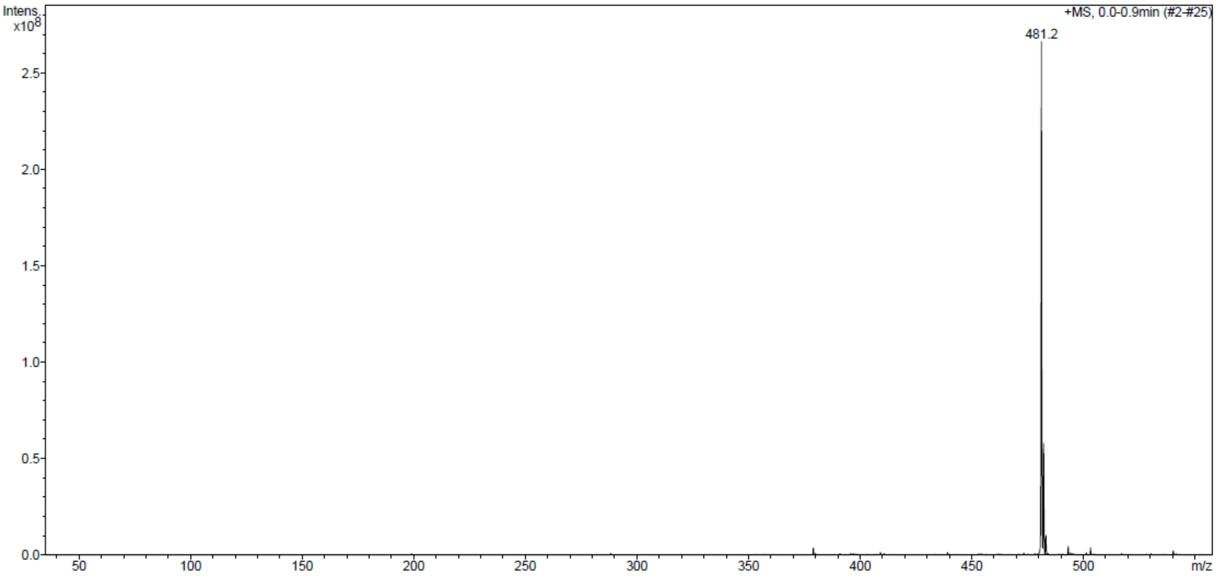

Bruker Daltonics DataAnalysis 3.1 printed: 09/27/19 10:27:24 Page 1 of 1

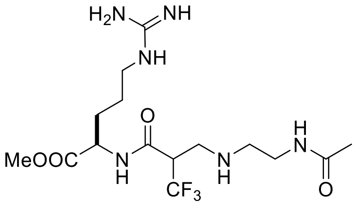
H2N
NH
NH
O
MeOOC
N
H
CF3
N
H
H
N
O

16

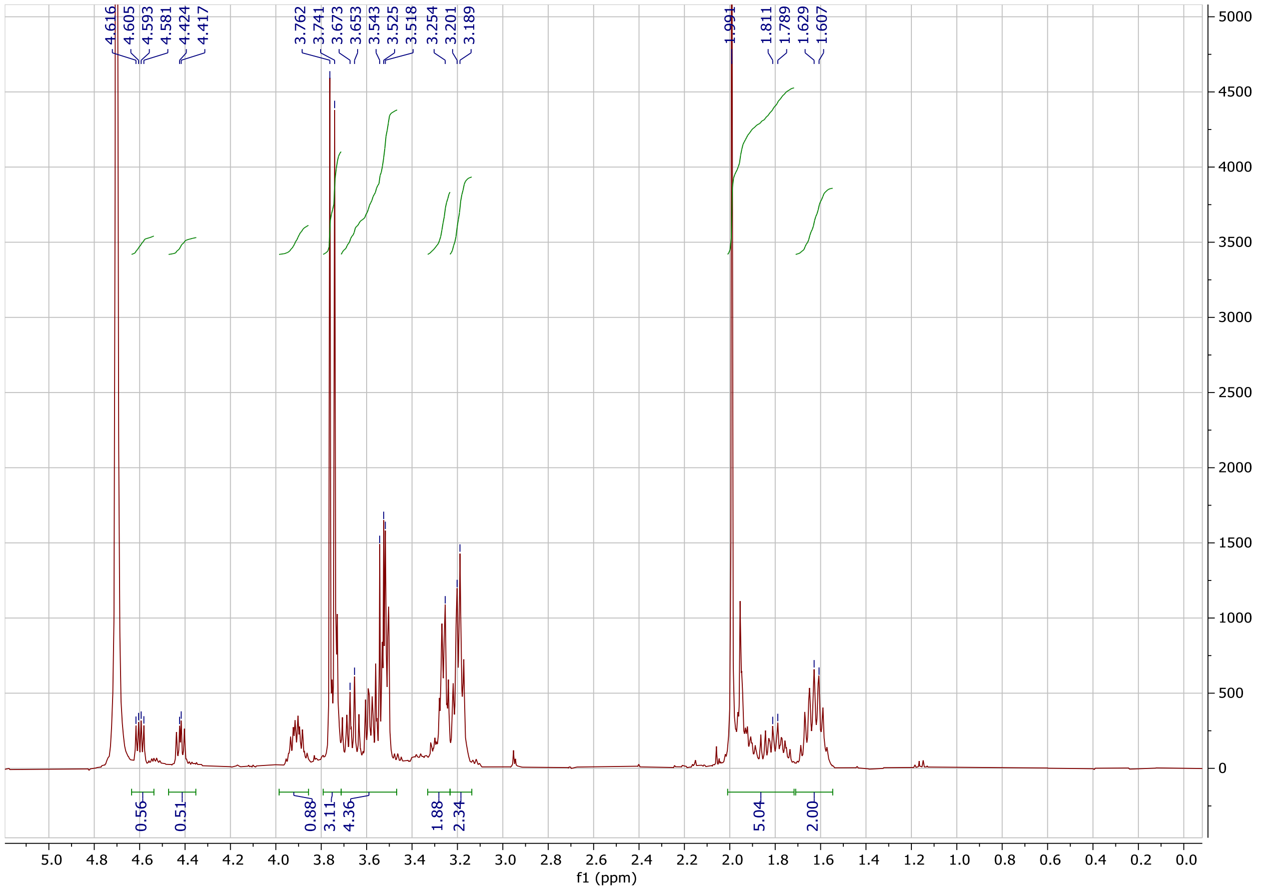
4.616
4.605
4.593
4.581
4.424
4.417
3.762
3.741
3.673
3.653
3.543
3.525
3.518
3.254
3.201
3.189
1.991
1.811
1.789
1.629
1.607
0.56
0.51
0.88
3.11
4.36
1.88
2.34
5.04
2.00
f1 (ppm)

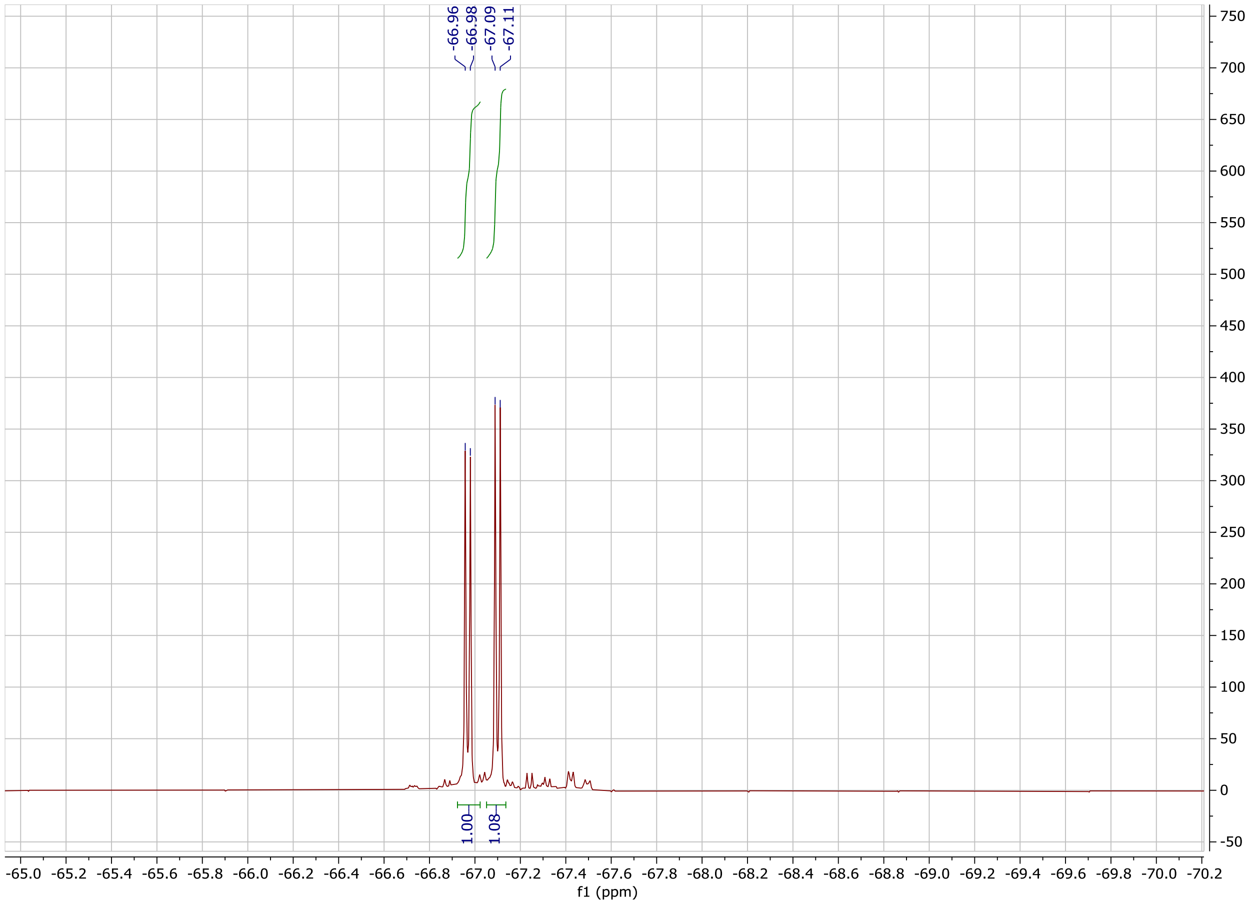

-L.G.S. - Laboratorio Grandi Strumenti - Display Report

Analysis Name	av elp329.d	Acquisition Date	07/28/20 14:35:48	Operator	Administrator
Sample Name		Method	Copy of _01tmix_posneg lm.MS	Instrument	esquire3000plus
Comment	1mg/ml dil 1:100 MeOH<br>Richiedente: Lopresti				

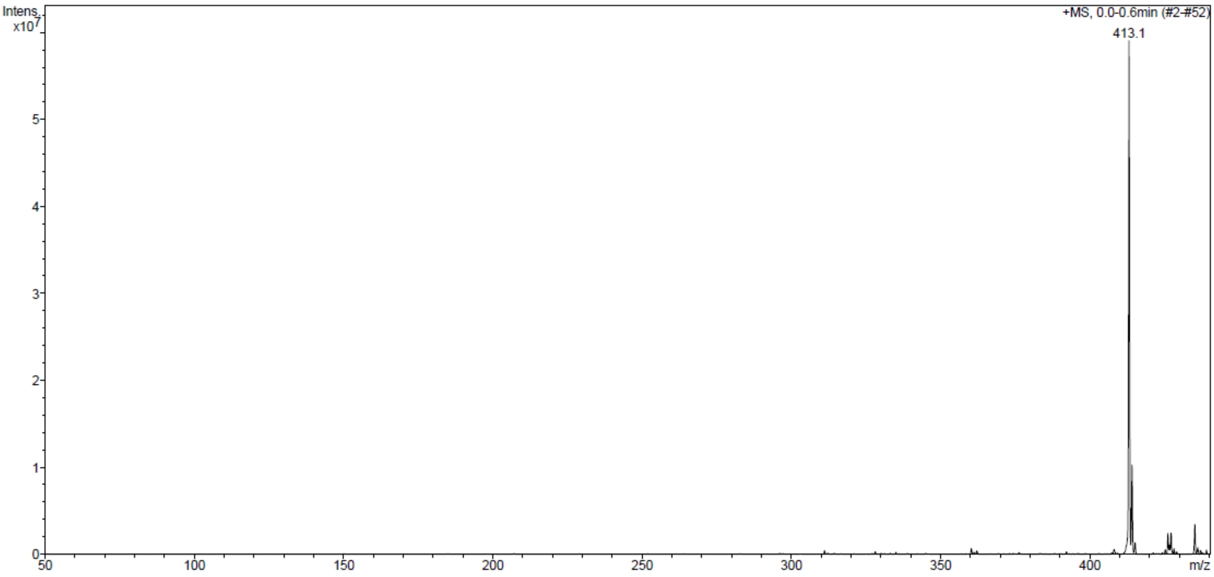

Bruker Daltonics DataAnalysis 3.1 printed: 07/28/20 14:37:00 Page 1 of 1

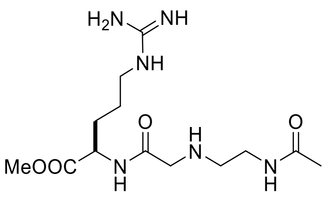

17

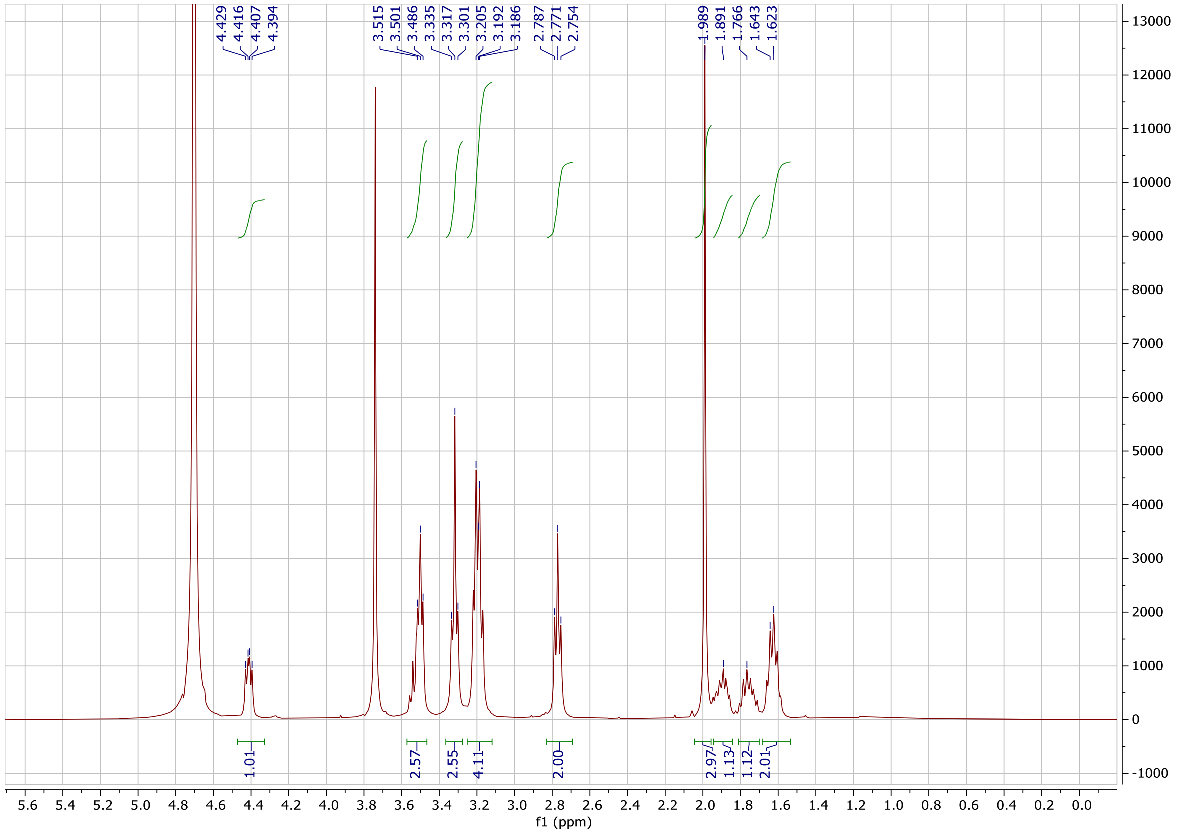

-L.G.S. - Laboratorio Grandi Strumenti - Display Report

Analysis Name	av elp338.d	Acquisition Date	07/28/20 11:10:29	Operator	Administrator
Sample Name		Method	Copy of _01tmix_posneg lm.MS	Instrument	esquire3000plus
Comment	1mg/ml dil 1:100 MeOH<br>Richiedente: Lopresti				

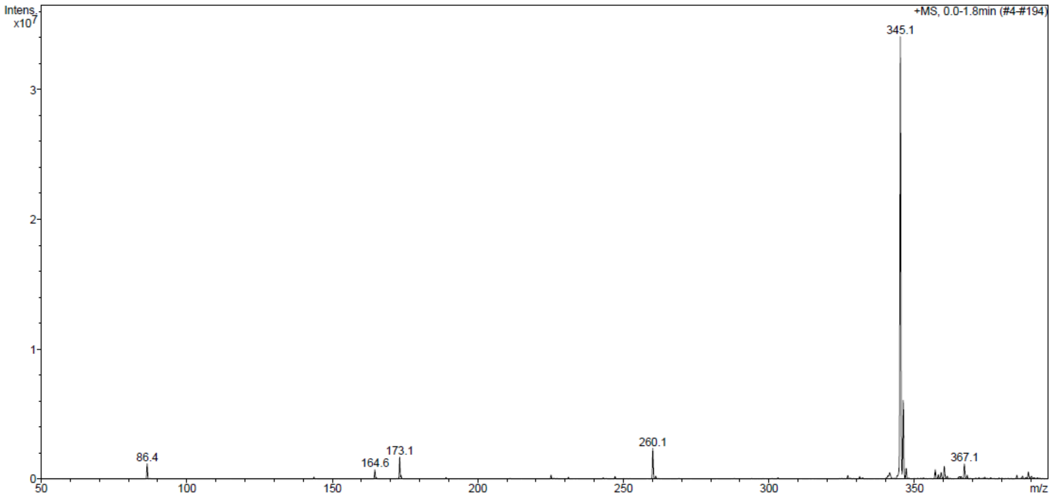

Bruker Daltonics DataAnalysis 3.1 printed: 07/28/20 11:12:44 Page 1 of 1

PAMAM G2–hfVal-Arg 1

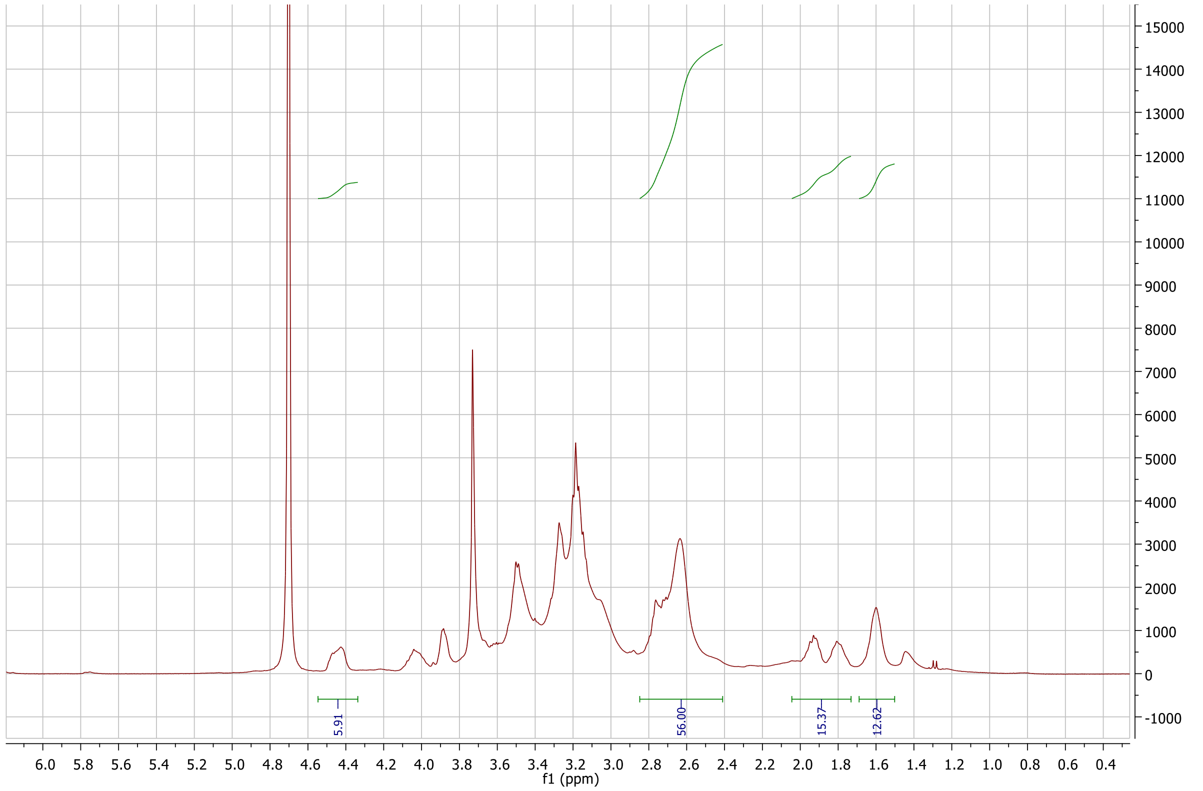

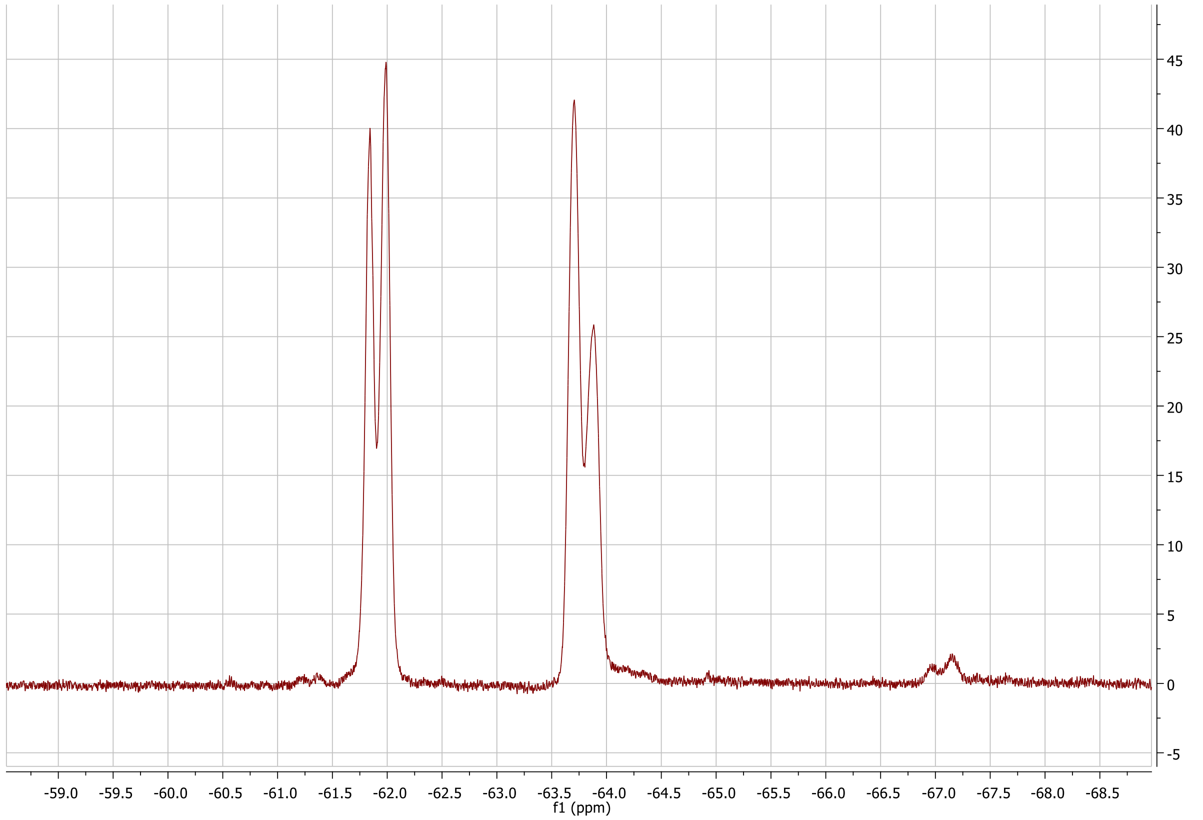

PAMAM G4–hfVal-Arg 2

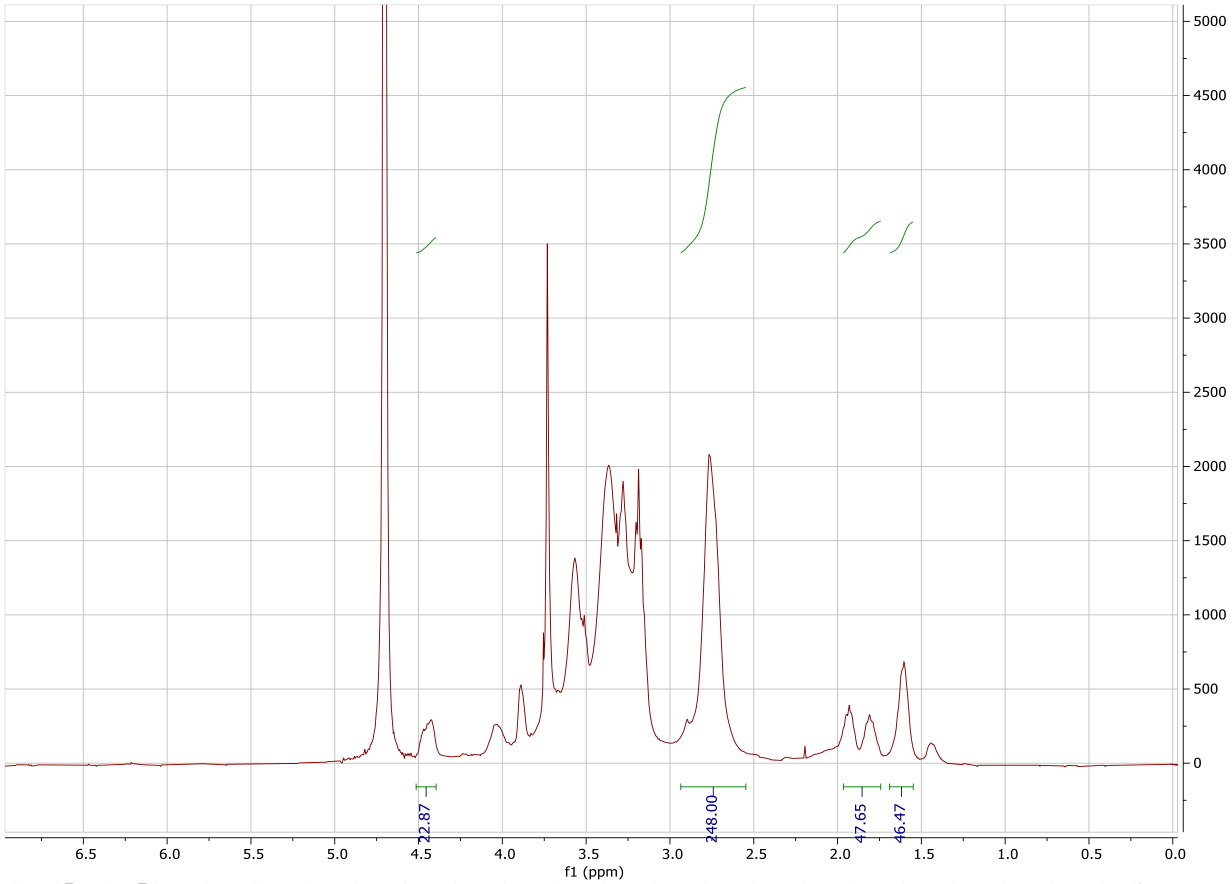

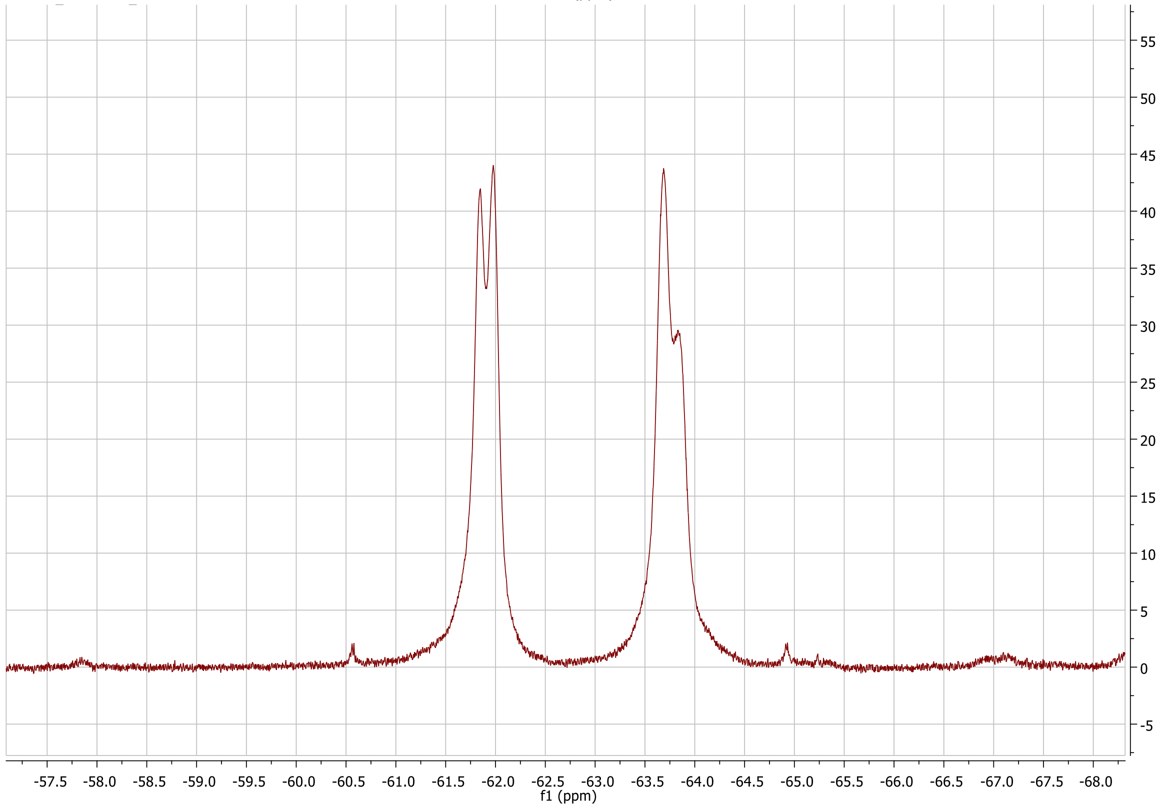

PAMAM G2-αtfm-βAla-Arg 3

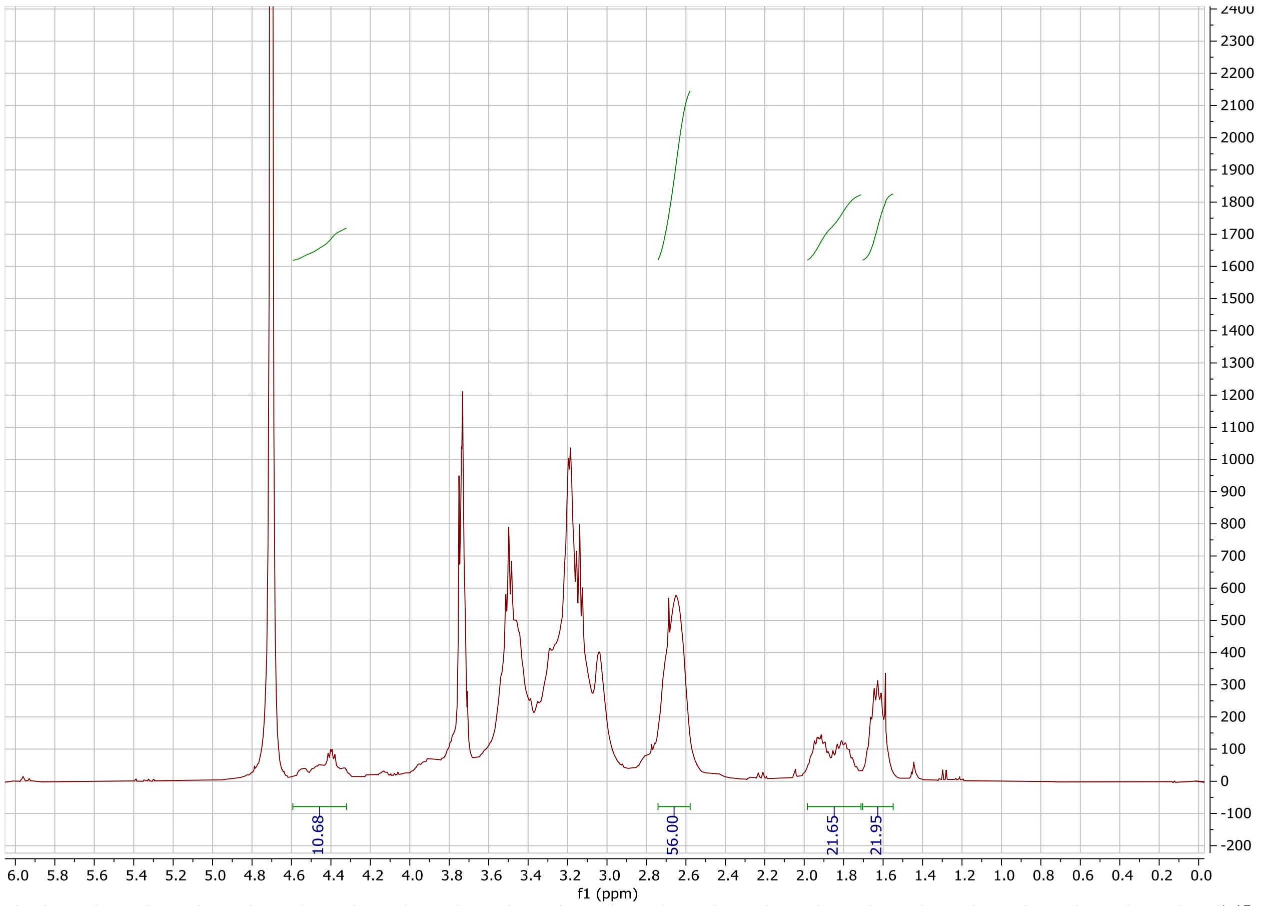

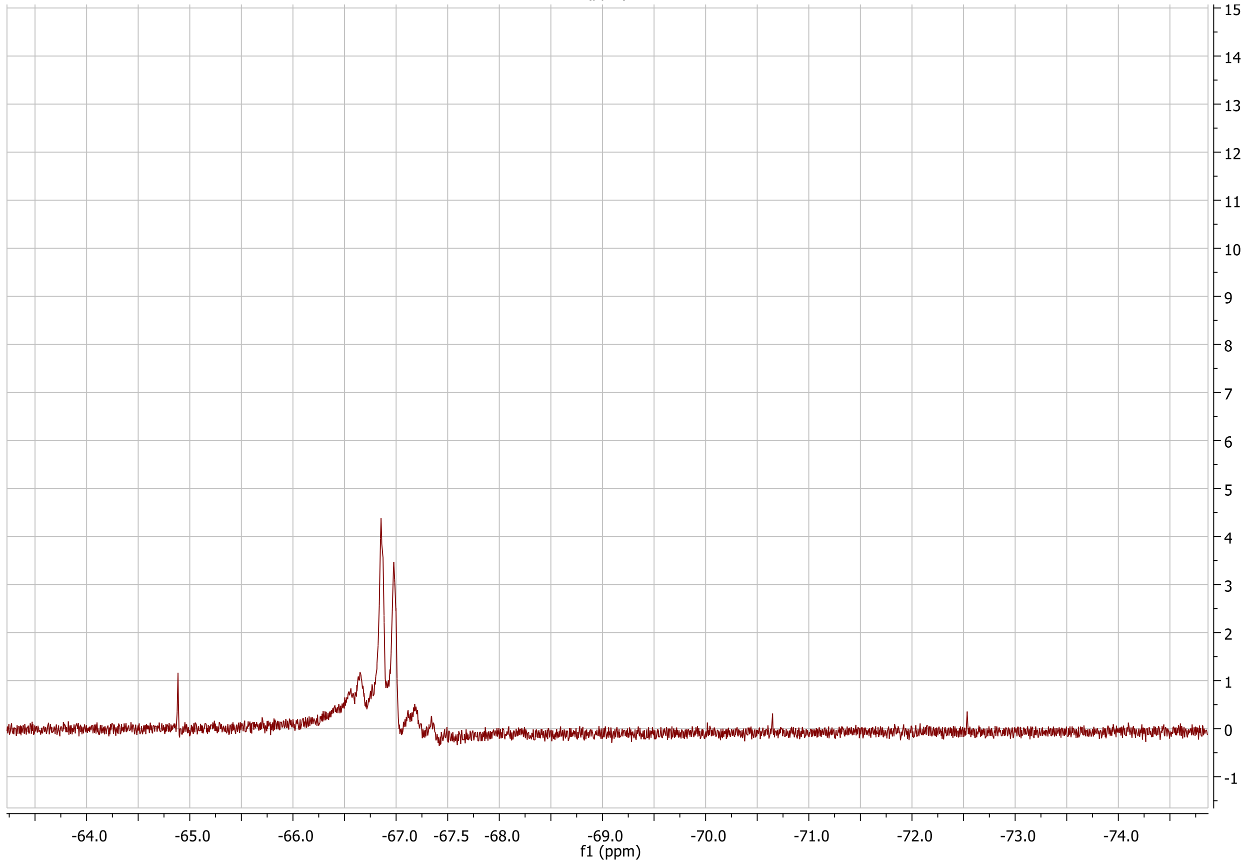

PAMAM G4-αtfm-βAla-Arg 4

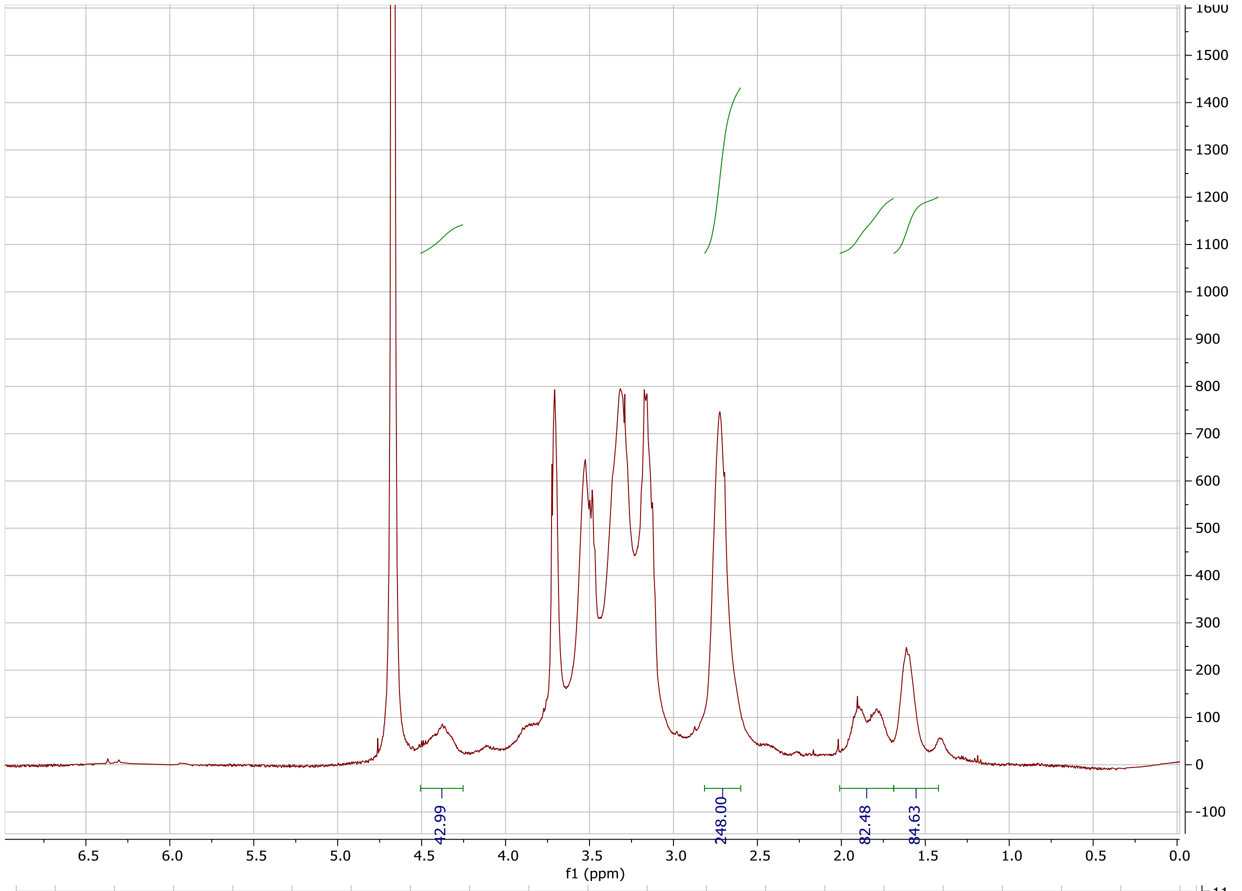

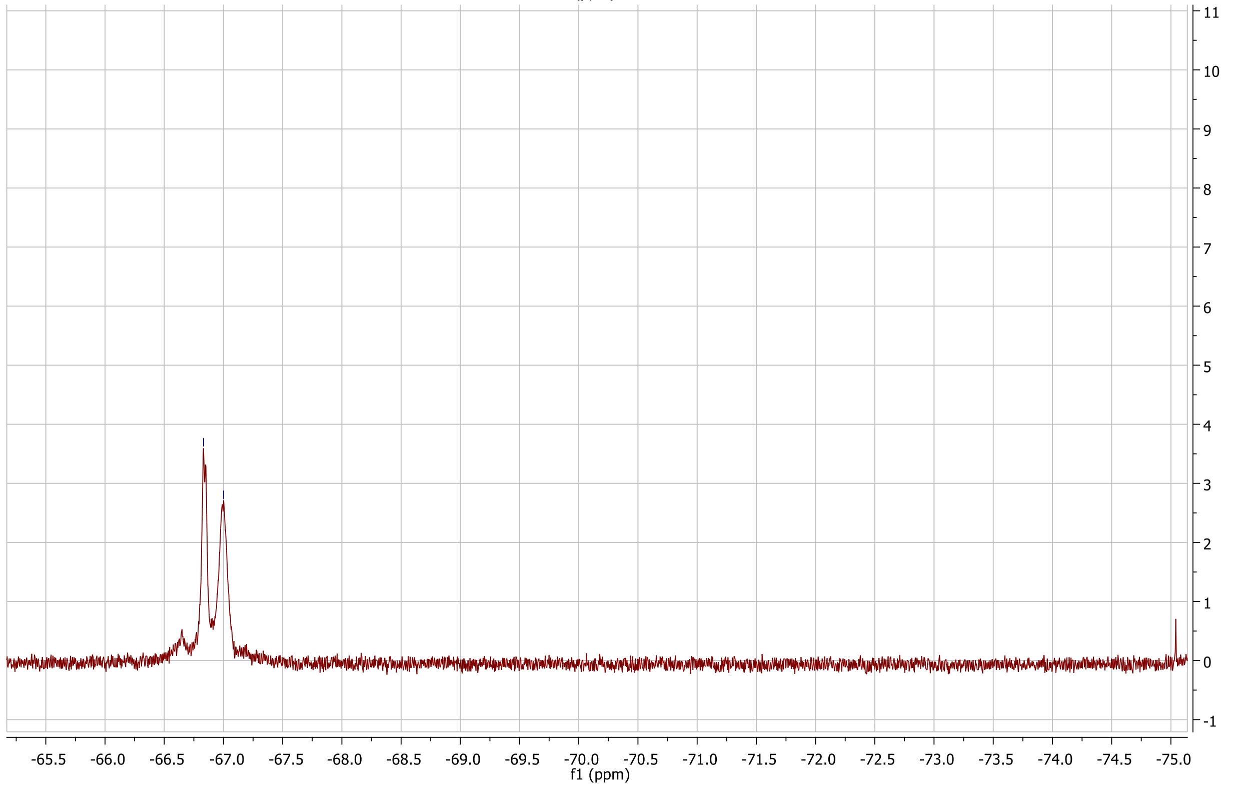

PAMAM G2 -βAla-Arg 13

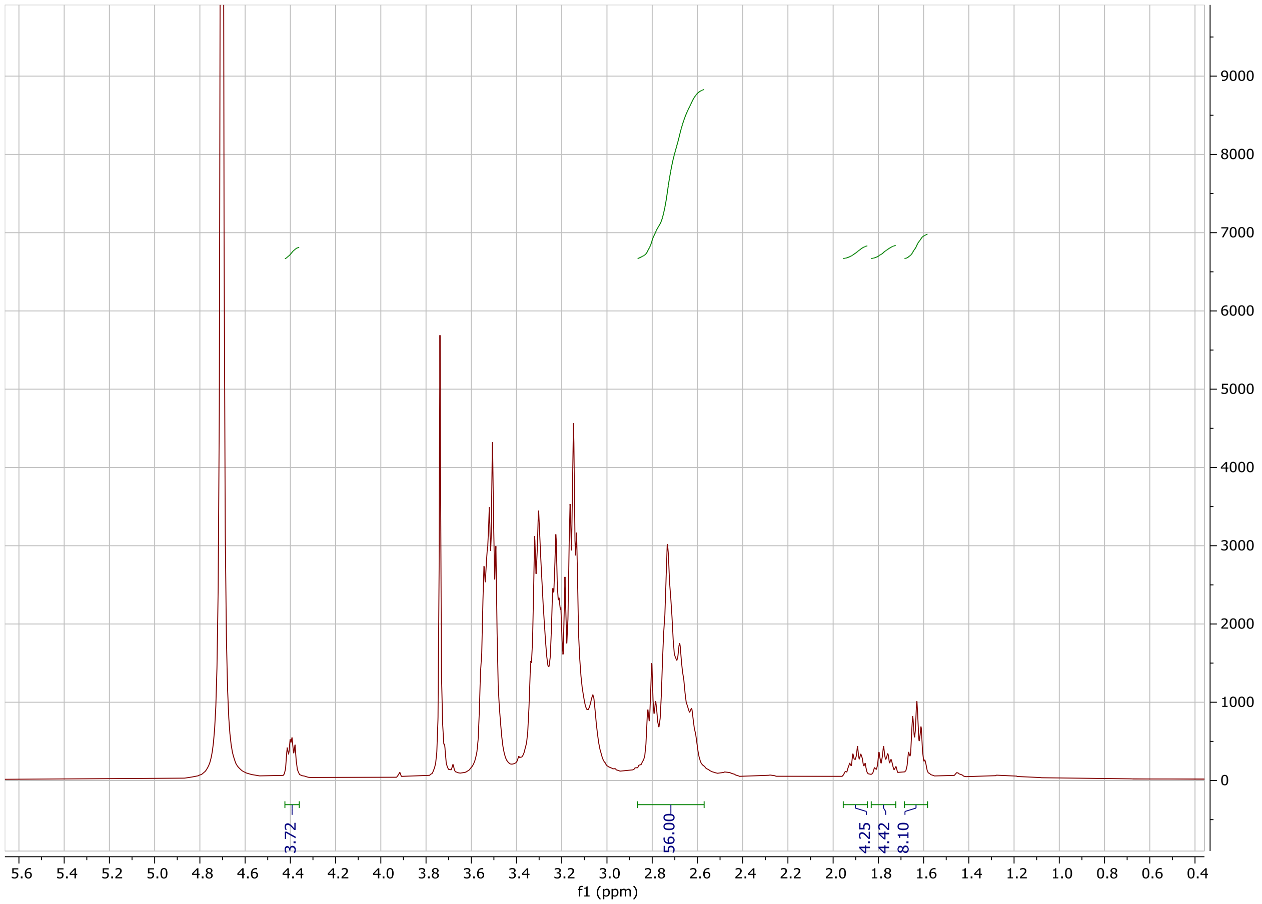

Figure S1. Agarose gel analysis of model DNA (Figure S1a) and pGFP (Figure S1b), used as preliminary complexation behavior analyses for PAMAM G4-αtfm-βAla-Arg **4** and PAMAM G2 dendrimers at different N/P ratios, namely 5, 15 and 30 each. The first lane corresponds to the DNA ladder, while in the last lane free herring sperm DNA or pGFP plasmid were loaded as negative controls.

PAMAM G4-αtfm-βAla-Arg **4** dendrimer at N/P ratio equal to 5 do not complex both model DNA and pGFP, but at higher N/P ratio a good complexation is observed. This is confirmed by gel retardation assay, in which free DNA runs through the gel with a stained nucleic acid intensity decreasing by increasing polymer concentration. Moreover, at higher N/P ratios formed complexes remain stuck in the loading wells. On the other hand, the low generation PAMAM G2 can form complexes with both model DNA and plasmid at all tested N/P ratios, as demonstrated by no detectable signals of nucleic acids bands through the gel but stuck in the loading wells.

Free pGFP plasmid shows a main band at 2.7 kb and two more secondary bands at higher weights (6 kb and >10 kb, respectively). These bands do not correspond to real nucleic acid dimension as it has not been linearized nor fragmented; these bands correspond to the circular plasmid isoforms assumed during gel migration.

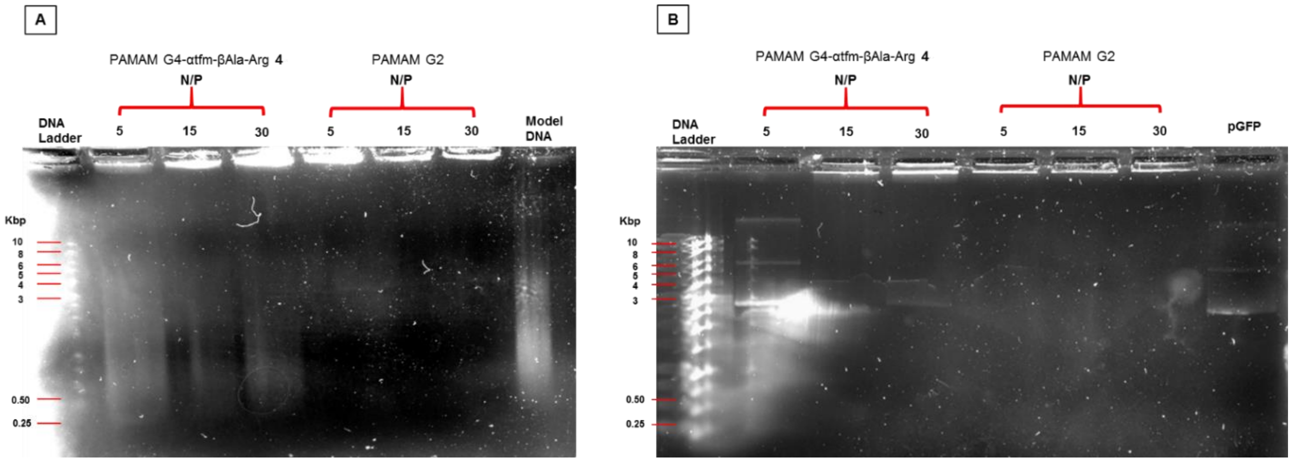

Figure S2. Particle size distribution by intensity (black line), volume (red line) and number (blue line) for the four dendriplexes synthesized: a) PAMAM G2-hfVal-Arg **1**, b) PAMAM G4-hfVal-Arg **2**, c) PAMAM G2-αtfm-βAla-Arg **3**, d) PAMAM G4-αtfm-βAla-Arg **4**

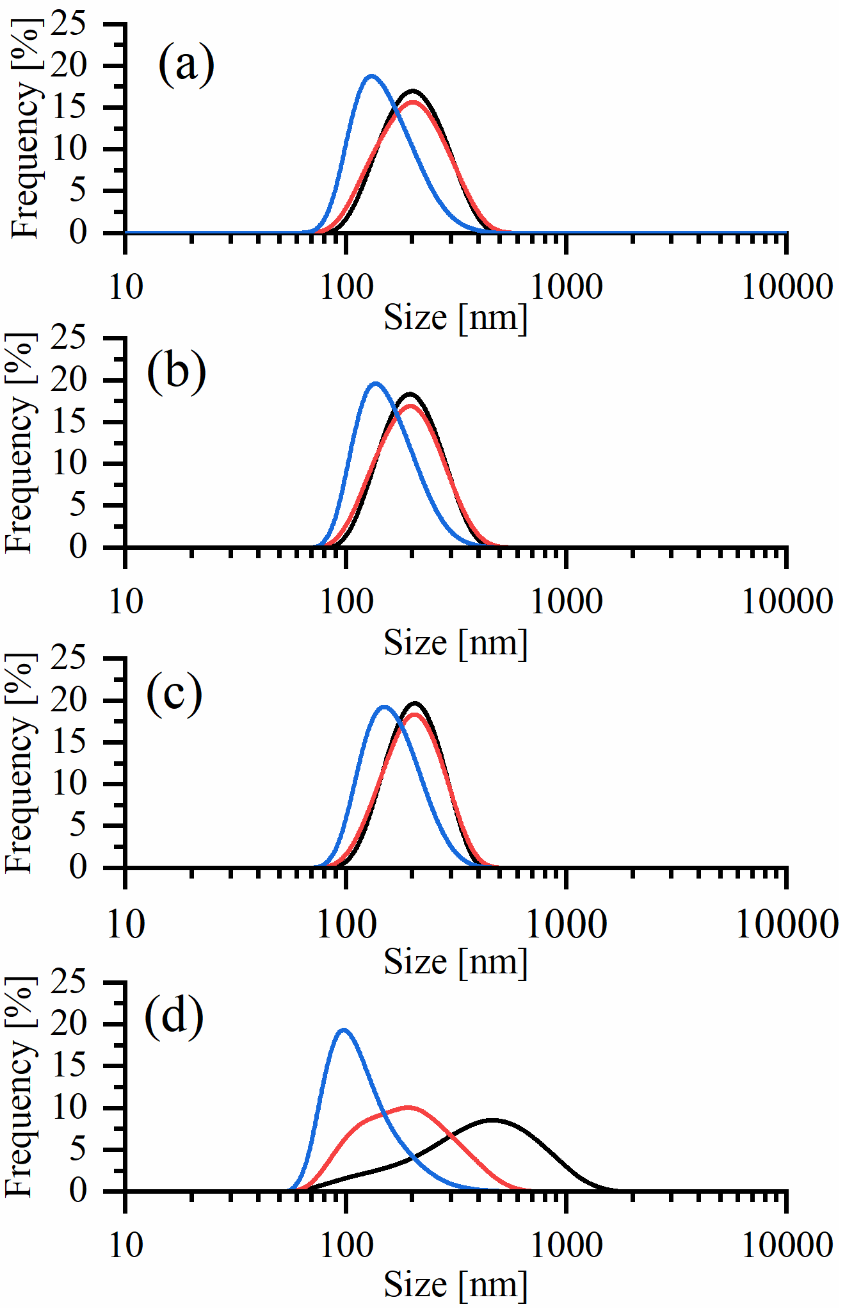

Figure S3. In the graphs below, the Relative Transfection Efficiencies of each tested system at N/P ratio equal to 15 are reported. Single TEs have been normalized by the TE of the positive control bPEI 25kDa. Relative TEs of undecorated PAMAM G2 and G4 dendrimers are reported, underlying a very low transfection efficiency of these systems, although some capability of forming complexes with DNA, as demonstrated by gel retardation assays. On the other hand, the TEs of our proposed systems, resulting in a very depicting the remarkable transfection efficiency of PAMAM G2-hfVal-Arg 1 and PAMAM G4-hfVal-Arg 2 dendrimers, with six-fold and four-fold higher TEs respectively as compared to both the positive control bPEI 25kDa (fixed to 1), and the undecorated PAMAM G2 and PAMAM G4 dendrimers.

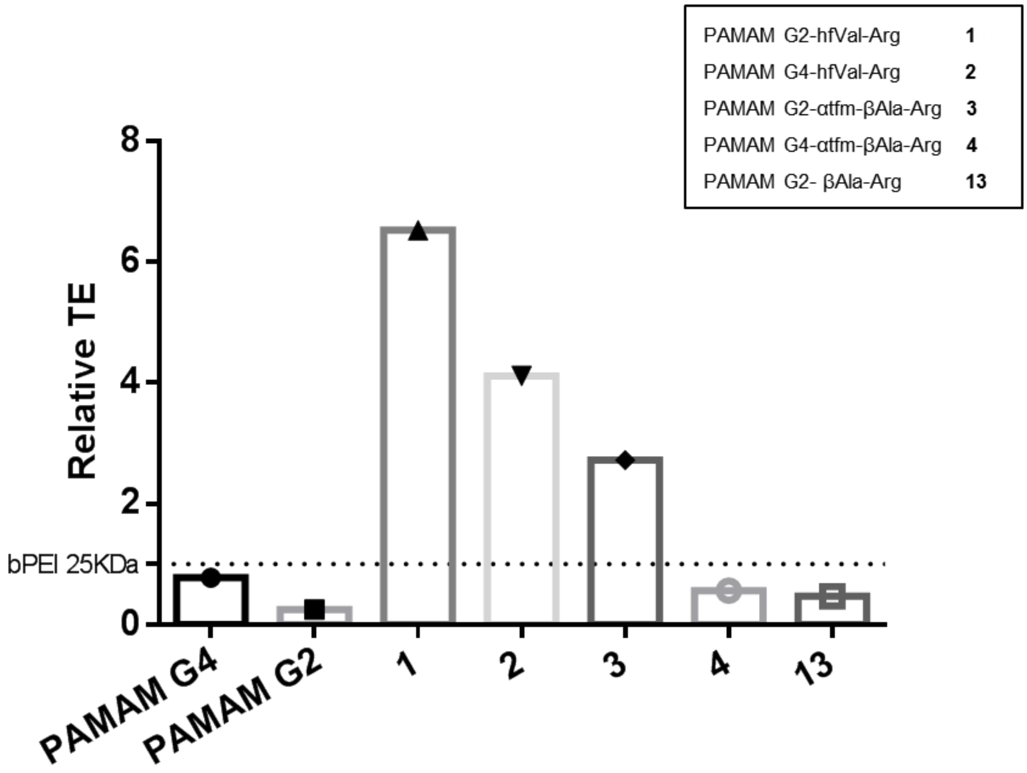

Supplement: Supplementary file 1 — bc3c00139_si_001.pdf [file bc3c00139_si_001.pdf]
